# Uncertainty in the impact of liver support systems in acute-on-chronic liver failure: a systematic review and network meta-analysis

**DOI:** 10.1186/s13613-020-00795-0

**Published:** 2021-01-18

**Authors:** Klementina Ocskay, Anna Kanjo, Noémi Gede, Zsolt Szakács, Gabriella Pár, Bálint Erőss, Jan Stange, Steffen Mitzner, Péter Hegyi, Zsolt Molnár

**Affiliations:** 1 Institute for Translational Medicine, Medical School, University of Pécs, Szigeti út 12. 2nd floor, Pécs, 7624 Hungary; 2 Heim Pál National Paediatric Institute, Budapest, Hungary; 3 Institute of Bioanalysis, Medical School, University of Pécs, Pécs, Hungary; 4 Division of Gastroenterology, First Department of Medicine, Medical School, University of Pécs, Pécs, Hungary; 5 Division of Nephrology, Department of Medicine, University of Rostock, Rostock, Germany; 6 First Department of Medicine, University of Szeged, Szeged, Hungary; 7 Translational Medicine Foundation, Szeged, Hungary; 8 Department of Anaesthesiology and Intensive Therapy, Faculty of Medicine, Poznan University of Medical Sciences, Poznan, Poland

**Keywords:** Network meta-analysis, Liver support therapy, Overall survival, Transplant-free survival, SUCRA, Plasma exchange, ELAD, MARS, Prometheus, BioLogic-DT

## Abstract

**Background:**

The role of artificial and bioartificial liver support systems in acute-on-chronic liver failure (ACLF) is still controversial. We aimed to perform the first network meta-analysis comparing and ranking different liver support systems and standard medical therapy (SMT) in patients with ACLF.

**Methods:**

The study protocol was registered with PROSPERO (CRD42020155850). A systematic search was conducted in five databases. We conducted a Bayesian network meta-analysis of randomized controlled trials assessing the effect of artificial or bioartificial liver support systems on survival in patients with ACLF. Ranking was performed by calculating the surface under cumulative ranking (SUCRA) curve values. The RoB2 tool and a modified GRADE approach were used for the assessment of the risk of bias and quality of evidence (QE).

**Results:**

In the quantitative synthesis 16 trials were included, using MARS®, Prometheus®, ELAD®, plasma exchange (PE) and BioLogic-DT®. Overall (OS) and transplant-free (TFS) survival were assessed at 1 and 3 months. PE significantly improved 3-month OS compared to SMT (RR 0.74, CrI: 0.6–0.94) and ranked first on the cumulative ranking curves for both OS outcomes (SUCRA: 86% at 3 months; 77% at 1 month) and 3-month TFS (SUCRA: 87%) and second after ELAD for 1-month TFS (SUCRA: 76%). Other comparisons did not reach statistical significance. QE was moderate for PE concerning 1-month OS and both TFS outcomes. Other results were of very low certainty.

**Conclusion:**

PE seems to be the best currently available liver support therapy in ACLF regarding 3-month OS. Based on the low QE, randomized trials are needed to confirm our findings for already existing options and to introduce new devices.

## Introduction

Acute-on-chronic liver failure (ACLF) is a clinical syndrome defined by the acute deterioration of chronic liver disease and the rapid development of organ failures, associated with high short-term mortality.

ACLF is due to exogenous and endogenous precipitating factors called pathogen- and damage-associated molecular patterns (PAMPs and DAMPs) [1, 2]. The release of these molecules by necrosis or infection triggers an excessive inflammatory response, resulting in organ failures. Most patients developing ACLF have preexisting cirrhosis, which is in itself a hyperinflammatory state [3, 4]. Another aggravating factor is the immune paralysis described by several studies [5–9], which prevents effective countermeasures against infection and makes patients prone to serious infective complications.

Several therapies have been tested for the replacement of hepatic functions. So far, liver transplantation is the only curative therapy available. Survival rates are good, but availability and eligibility for transplant in ACLF differs by country [10]. In the CANONIC study, only 4.5% of ACLF patients received transplant. Reportedly, low transplant rates are due to the high prevalence of infection and organ failure. Waiting-list mortality exceeds 50% in this population [10].

The development of extracorporeal liver support systems dates back to the seventies with the aim to stabilize patients at the time of acute decompensation when transplant is not available or bridge patients to transplant [11]. At first, these devices were designed to replace only excretory functions and were based on hemoperfusion and adsorption [12]. The newer technologies combined these methods with bioreactors containing hepatocytes creating bioartificial liver support systems with the potential of synthetic activity.

The Asian Pacific Association for the Study of the Liver (APASL) consensus guideline from 2019 states that “plasma exchange appears to be a promising and effective bridging therapy in patients with ACLF to liver transplant or spontaneous regeneration [1, C]” [13]. The European Association for the Study of the Liver (EASL) Clinical Practice Guidelines do not recommend liver support therapies for the treatment of ACLF, but underline the importance of further studies, because in specific subgroups ACLF seems beneficial [14].

Numerous pairwise meta-analyses of randomized controlled trials (RCTs) have been published assessing short-, middle-, and long-term survival benefit of liver support therapies with controversial results [15–22]. These meta-analyses faced serious limitations, as they pooled together data from studies testing different devices, in some cases with different follow-up lengths. A network meta-analysis (NMA), on the other hand, can handle multiple interventions and rank them, if the assumption of transitivity is met [23].

To facilitate international discussion and consensus, we decided to perform the first NMA comparing all available and tested liver support systems to each other and standard medical therapy (SMT) in patients with ACLF and ranking these treatments by survival benefit.

## Methods and materials

The protocol for this review was registered in the PROSPERO database under registration number CRD42020155850. There were no protocol deviations. This meta-analysis was reported according to The PRISMA Extension Statement for Reporting of Systematic Reviews Incorporating Network Meta-analyses of Health Care Interventions (PRISMA-NMA) [24].

### Eligibility criteria

Parallel randomized controlled trials assessing the safety and efficacy of artificial and bioartificial liver support therapies in adult patients with acute-on-chronic liver failure (ACLF) were eligible for inclusion, regardless of the current availability of the tested therapy and length of follow-up. Conference abstracts were included to reduce publication bias. Crossover studies were excluded from the analyses of survival due to concerns about the carryover effect, but were included in the systematic review. ACLF definitions used in the included RCTs were accepted, as there is a lack of international consensus regarding this matter. For the studies published before ACLF was introduced as a clinical entity, the review authors decided eligibility based on the eligibility criteria used in the study. Due to substantial heterogeneity regarding the definitions or the timing of measurements, some outcomes were included only in the qualitative synthesis. Studies with shorter or longer follow-up periods than the assessed outcomes were also included in the systematic review.

### Search strategy and selection

The systematic search was conducted up to the 15th December 2019 in the following databases: MEDLINE (via PubMed), Embase, CENTRAL, Web of Science, and Scopus, with the search key designed based on the PICO format––(“hepatic failure” OR “liver failure” OR “end-stage liver disease” OR “cirrhosis” OR “alcoholic hepatitis”) AND (“liver support system” OR “liver support device” OR “liver assist device” OR “artificial liver” OR “bioartificial liver” OR “extracorporeal liver” OR “albumin dialysis” OR “extracorporeal cellular therapy” OR “MARS” OR “Prometheus” OR “fractioned plasma separation and adsorption” OR “hemoadsorption”) AND random*. No filters or restrictions were applied. References of included studies, citing articles, and authors’ accessible publications in a search engine (Google Scholar) and ResearchGate were hand searched for further eligible publications.

### Data extraction

Data extraction was performed by two independent investigators (KO and AK) in duplicate using Endnote X9, Clarivate Analytics and Windows Excel 2016, Microsoft. In the case of discrepancies, agreement was reached by two experts (ZM or ZS). As a measure of inter-rater reliability, Cohen’s kappa coefficients (*κ*) for the selection of abstracts and full texts were counted. Information collected from each study and additional information used are detailed in Additional file 1.

### Risk of bias assessment and quality of evidence

The risk of bias assessment was conducted in duplicate using Version 2 of the Cochrane risk-of-bias tool for randomized trials (RoB 2) for overall and transplant-free survival separately [25].

For the four outcomes assessed in the NMA, quality of evidence was assessed in duplicate according to the Grades of Recommendation, Assessment, Development and Evaluation Working Group’s recommendations, using a modified GRADE approach [26].

### Statistical analysis

A Bayesian method was used to perform pairwise meta-analyses and NMAs with the random effect model for overall survival (OS) and transplant-free survival (TFS). For the analysis of transplant-free survival, transplant counted as an event similar to death. In case no patient received liver transplantation, OS and TFS were identical. If available, data for the intention-to-treat population were used.

We used risk ratios (RR) for dichotomous data with 95% credible intervals (95% CrI). We optimized the model and generated posterior samples using the Monte-Carlo methods running in four chains. We set at least 20,000 adaptation iterations to get convergence and 10,000 simulation iterations. Network estimates (pooled direct and indirect data) of each intervention compared to standard medical therapy and other interventions are presented in forest plots, summarized in a league table (as shown in the results section). We were unable to use the node-splitting analysis to examine the consistency assumption because of the star-shaped configuration of the networks [27]. We ranked the interventions by their posterior probability by calculating the surface under cumulative ranking (SUCRA) curve values ranging from 0 to 100%. The higher the SUCRA value, and the closer to 100%, the higher the likelihood that a therapy is in the top rank or one of the top ranks; the closer to 0 the SUCRA value, the more likely that a therapy is in the bottom rank, or one of the bottom ranks [28]. We also provided rankograms, showing the probability of achieving certain ranks. Frequentist comparison-adjusted funnel plots were created for 1- and 3-month OS, and Egger's tests were performed to assess small-study effect. The low number of studies in the TFS analyses did not enable this method. In an additional analysis, methodology-based evaluation was performed. All calculations were performed with R (V. 3.5.2) package gemtc (V. 0.8–2) along with the Markov Chain Monte Carlo engine JAGS (V. 3.4.0) and STATA 16.0 (StataCorp LLC).

## Results

### Search and selection

**Fig. 1:**
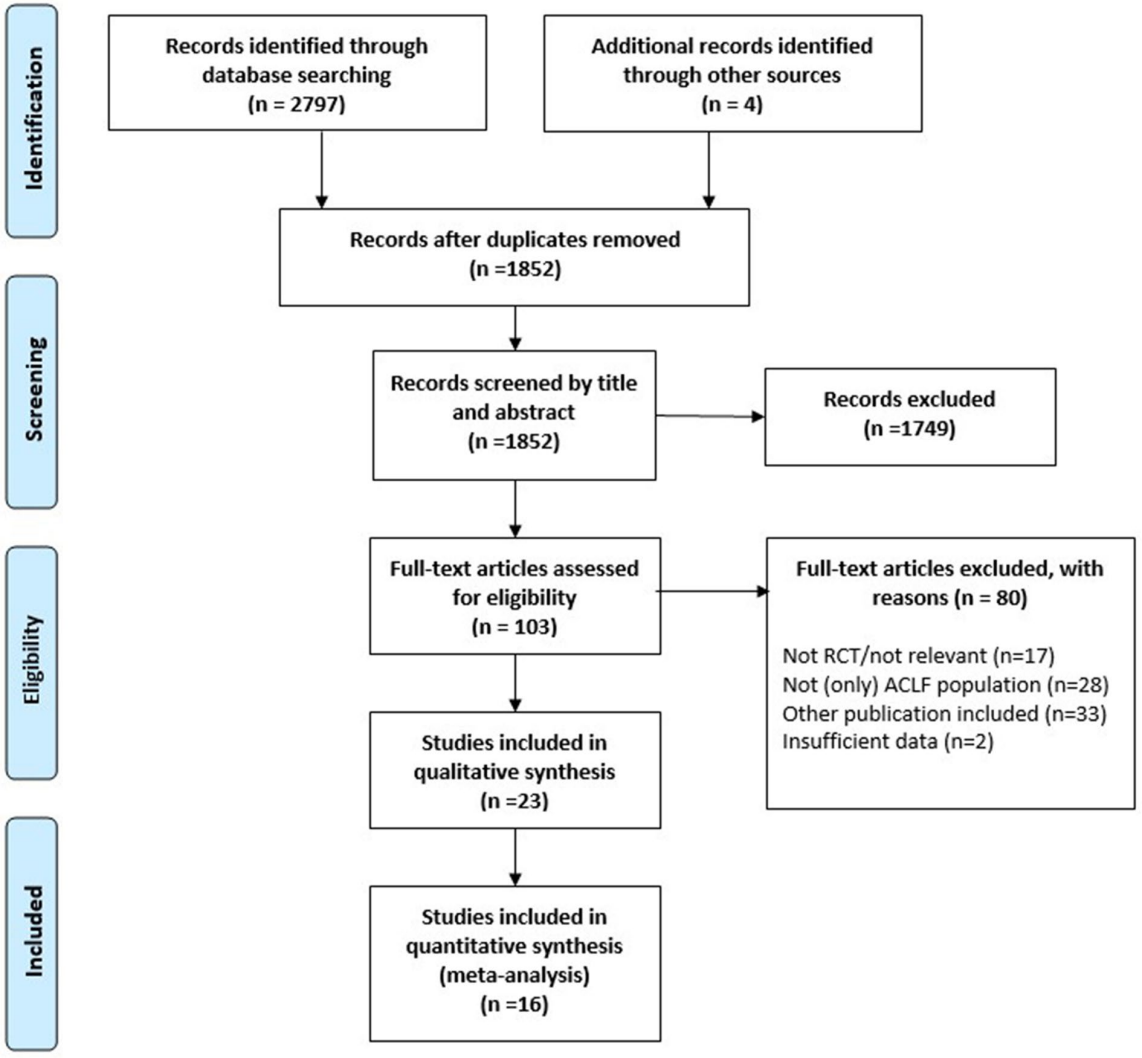
Flowchart of study selection according to the PRISMA Statement

The systematic search yielded 2797 records. Four additional articles were identified through manual search and from previous meta-analyses. *κ* for abstracts and full texts was 0.87 and 0.90, respectively, marking almost perfect agreement in both cases. One hundred three full texts were assessed for eligibility. Twenty-three articles proved to meet the eligibility criteria for the systematic review and 16 were included in the data synthesis (Fig. 1).

### Characteristics of the included studies

The main characteristics of the 23 eligible studies included in qualitative synthesis are shown in Table 1. Of the 16 studies, enrolling 1670 patients included in the meta-analysis, 15 compared a type of artificial [29–38] or bioartificial [39–43] liver support system to standard medical therapy and one study compared MARS versus MARS plus plasma exchange [44]. The most common etiologies of underlying diseases were viral infection and alcohol. From the 1526 participants with available information on gender, 1064 were males (69.8%). ACLF definitions, eligibility criteria, baseline characteristics, and outcomes of the individual studies are reported in Table 1.

**Table 1:** Characteristics of included studies

First author, publication year	Eligibility criteria	Etiology of the underlying disease and baseline characteristics	Intervention(s) and/or comparison	Outcomes
Banares (2013)	Inclusion: presumptive diagnosis of cirrhosis with an identifiable triggering event; an increase of TBIL > 5 mg/dl and at least one of the following: HRS, HE ≥ grade II, rapidly progressive hyperbilirubinemia (> 50% increase from TBIL levels at admission) > 20 mg/dl at admission Exclusion: progressive jaundice as a consequence of the natural course of cirrhosis; extrahepatic cholestasis; PLT < 50,000/mm^3^; INR > 2.3; suspected or evident DIC; need for RRT; intrinsic renal disease; uncontrolled infection; active bleeding; HCC > 4 cm in diameter; portal vein thrombosis; severe cardiopulmonary disease; MAP < 60 mmHg despite vasopressor therapy; major surgical procedure within the last 4 weeks; HIV infection	Mostly alcoholic; viral, autoimmune, drug-induced, NASH, etc. Age (years)^a^: 51.8/50.0 Males (%): 66.7/70.8 MELD^a^:25.6/24.1	MARS/SMT	Survival, HE, laboratory parameters, AEs
Duan (2018)	Inclusion: 15–65 years; clinical diagnosis of ACLF; obvious gastrointestinal and/or systemic toxic symptoms; TBIL > 5 times upper limit of normal or daily increase > 1 mg/dl; prothrombin activity of 10–50%; INR 1.6–4.0, or prothrombin time > 5 s longer than the control but < 20 s, HE absent or grade I–II; no or mild ascites/pleural effusion Exclusion: primary or metastatic liver cancer; uncontrolled severe infection; shock; active bleeding within 3 days; grade III–IV HE; PLT < 40 × 10^9^/l; creatinine > 1.5 mg/ml; severe esophageal varices	Mostly alcoholic and viral; drug-induced, autoimmune, unknown, “acute/subacute” Age^a^ (years): 39.5/39.2 Males (%): 96.9/88.2 MELD^a^: 28.0/30.8	ELAD/SMT	Survival, AEs
Ellis (1999)	Inclusion: acute alcoholic hepatitis, HE ≥ grade II Exclusion: pregnancy; MAP < 50 mmHg despite adequate volume loading and appropriate use of inotropes; respiratory failure; cerebrovascular event within the previous 12 months, a recent upper gastrointestinal hemorrhage; poorly controlled epilepsy; recent myocardial infarction/ischemia	Alcoholic Age^b^ (years): 46/43 Males (%): 60/80 MELD/CTP: NR	BioLogic-DT/SMT	Survival, HE, physical and laboratory parameters, AEs
Hassanein (2007)	Inclusion: ≥ 18 years, manifestations of cirrhosis and HE grade III–IV Exclusion: active hemorrhage; hemodynamic instability; acute cardiopulmonary complications (pulmonary edema, massive aspiration pneumonia, heart failure); pregnancy; active RRT; drug intoxication or irreversible brain damage or nonhepatic causes of altered mental status; acute liver failure; HCC; received transplant	Mostly alcoholic or viral; autoimmune, drug induced, unknown Age^b^ (years): 49/56 Males (%): 61.5/48.4 MELD^b^: 33/38	MARS/SMT	HE, AEs, laboratory parameters (survival is additional)
Heemann (2002)	Inclusion: 18–65 years; cirrhosis (CTP ≥ 7) and a superimposed acute liver injury leading to decompensation and severe hyperbilirubinemia (TBIL ≥ 20 mg/dl) Exclusion: hepatobiliary obstruction; active bleeding or sepsis causing hemodynamic instability; comorbid conditions associated with a poor outcome; coma of nonhepatic origin; extensive surgery 30 days preceding admission; HRS; pregnancy	Mostly alcoholic; viral, drug induced Age^b^ (years): 48/57 Males (%): 50/63.6 CTP^b^: 11.5/12	MARS/SMT	Survival, HE, AEs, laboratory parameters
Hillebrand (2010)	Inclusion: acute decompensation of cirrhosis; SOFA score ≥ 9; and either a MELD score of ≥ 32, or MELD ≥ 24 and at least one of HE grade III–IV or type I HRS Exclusion: NR	Etiology, age, sex NR MELD^a^: 34.3/40.8	ELAD/SMT	Survival, AEs
Huang (2012)	Inclusion: chronic severe hepatitis B with HE ≥ grade II Exclusion: late stage disease; previous irreversible respiratory failure; severe brain edema with hernia; severe systemic circulation disorder accompanied by DIC; serious active bleeding	HBV Age^b^ (years): 43/42 Males (%): 78.3/75 MELD/CTP: NR	MARS ± PE	Survival, HE, AEs, laboratory parameters, cost of treatment
Kramer (2001)	Inclusion: documented cirrhosis and encephalopathy grade II or III had not improved with conventional treatment Exclusion: renal failure; hypotension (MAP < 55 mmHg); respiratory or multiorgan failure; fever of > 38.5 °C; bleeding requiring transfusion of > 2 units within the preceding 24 h; insulin-dependent diabetes mellitus; administration of sedatives within the preceding 2 days	Alcoholic, viral, autoimmune, unknown Age^b^ (years): 55/56 Males^c^ (%): 65% CTP^b^: 14/14.5	BioLogic-DT/SMT	HE, laboratory and physical parameters, AEs (survival is additional)
Kribben (2012)	Inclusion: 18–70 years; severe deterioration of chronic liver disease; CTP ≥ 10 (over 72 h); TBIL ≥ 5 mg/dl (over 72 h) Exclusion: pregnancy/lactation; HIV infection, intracranial bleeding; cerebrovascular disease; ARDS; circulatory shock with vasopressor therapy; persistent bleeding needing perfusion; chronic renal failure stage V; acute necrotizing pancreatitis; HCC, malignancy; INR > 3.0 or PLT < 30,000/l; extrahepatic cholestasis; liver resections or major hepatobiliary surgery in the previous 6 months except laparoscopic cholecystectomy; LT within 2 years, ALSS therapy within 7 days; participation in another clinical trial or this study priorly	Mostly alcoholic and viral; others not specified Age^a^ (years): 50/51 Males (%): 62/65 MELD^b^: 28/27	Prometheus/SMT	Survival, laboratory parameters; AEs
Mitzner (2000)	Inclusion: 18–60 years; HRS (serum creatinine > 1.5 mg/dl, oliguria < 500 ml/d, urine sodium < 20 mmol/l, central venous pressure > 8 cmH_2_O); need of hemodialysis/filtration treatment; chronic liver failure (3 of 4 criteria): ultrasonic signs of chronic damage or impaired synthesis function (hypoalbuminemia, 30 g/l, prolonged prothrombin time (quick value < 70%), AT III < 70%, serum cholinesterase < 40 umol/s/l) or hyperbilirubinemia (> 15 mg/dl) or grade III–IV HE Exclusion: fulminant hepatic failure; sepsis unresponsive to antibiotics; severe acute hemorrhages; malignancies; obstructive/chronic renal failure; pregnancy; severe cardiopulmonary disease	Mostly alcoholic; HBV, primary and secondary biliary cirrhosis Age^a^ (years): 49.6/43.8 Males (%): 37.5/40 CTP^a^: 12.5/12.2	MARS/SMT	Survival
Pyrsopoulos (2019)	Inclusion: SAH, age 18–50 years, total bilirubin ≥ 16 mg/dl, Maddrey score ≥ 32, not eligible for transplant Exclusion: PLT < 40,000/mm3; INR > 2.5; serum creatinine ≥ 1.3 mg/dl; MELD score ≥ 30; AST > 500 IU/l; infection unresponsive to antibiotics; reduction in TBIL ≥ 20% in the previous 72 h; hemodynamic instability; active bleeding; major hemorrhage; liver size reduction due to cirrhosis; occlusive portal vein thrombosis; bile duct obstruction; life expectancy of less than 3 months due to concomitant diseases; subject on hemodialysis; Wilson’s disease; NAFLD; Budd-Chiari syndrome; active viral hepatitis; pregnancy; received liver transplant	Alcoholic hepatitis Age^a^ (years): 39.1/39.5 Males (%): 60.3/60.3 MELD^a^: 24.8/25.6	ELAD/SMT	Survival, AEs
Qin (2014)	Inclusion: 18–70 years; presumptive diagnosis of chronic hepatitis B infection, HBV-associated cirrhosis, or hepatitis B surface antigen (HBsAg) carrier; rapidly progressive hyperbilirubinemia with TBIL > 10 mg/dl, within 28 days from symptom onset; INR > 1.5 or plasma prothrombin activity < 40% Exclusion: acute HBV infection; hepatitis E, A, D, or HIV superinfection; alcohol- or drug-induced liver injury; severe gastrointestinal bleeding; HCC; pregnancy	HBV Age^a^ (years): 44.1/48.7 Males (%): 82.7/72.3 MELD^a^: 28.6/29.5	PE/SMT	Survival, AEs
Sen (2004)	Inclusion: 18–75 years old; alcoholic liver disease; acute deterioration in liver function over 2–4 weeks leading to severe progressive clinical deterioration despite supportive care (over 48 h); jaundice (TBIL > 100 mol/l) and either HE Grade II or HRS; cirrhosis Exclusion: prior enrollment in another study; known hepatic/extrahepatic malignancy; uncontrolled infection or upper gastrointestinal bleeding over the previous 48 h; pregnancy; prior treatment with terlipressin for HRS; coexisting HIV infection; severe cardiorespiratory disease	Alcoholic Age^b^ (years): 45/44 Males (%): 78/67 MELD^b^: 16.5/19.4	MARS/SMT	Survival, HE, laboratory and physical parameters
Teperman (2012)	Inclusion: acute alcoholic hepatitis or acute decompensation of cirrhosis, MELD 18–35 Exclusion: NR	Alcoholic and not specified (baseline only given for PP subjects)	ELAD/SMT	Survival, time to progression, AEs
Thompson (2018)	Inclusion: ≥ 18 years, history of heavy alcohol abuse, maximum of 6 weeks between the last consumption, rapid onset of jaundice (TBIL ≥ 8 mg/dl), and coagulopathy (Maddrey's DF ≥ 32), stratum A: liver biopsy confirmed SAH/ 2 of the following: AST > ALT, leukocytosis, ascites stratum B: SAH + underlying chronic liver disease confirmed by biopsy, laboratory findings, and/or medical history Exclusion: end-stage cirrhosis; portal vein thrombosis; MELD > 35, PLT < 40,000/mm^3^; severe concomitant disease; uncontrolled bleeding; infection unresponsive to antibiotics; hemodynamic instability; chronic dialysis	Alcoholic hepatitis (superimposed or primary) Age^a^ (years): 46.5/44.8 Males%: 57.3/60.7 MELD^a^: 27.6/27.1	ELAD/SMT	Survival, laboratory parameters, AEs
Yu (2008)	Inclusion: acute-on-chronic hepatitis B liver failure (HBV-DNA ≥ 10,000 copies/mL); defined as severe jaundice (TBIL > 171 mmol/l), coagulopathy, and/or HE > grade II; previous lamivudine treatment; MELD > 30 Exclusion: obstructive and hemolytic jaundice; prolonged prothrombin time due to hematologic diseases; drug-induced hepatitis; Wilson's disease; alcoholic liver disease; autoimmune hepatitis; hepatitis C or D or HIV infection	HBV Age^a^ (years): 45.2/46.4 Males (%): 80/78.6 MELD^a,d^: 41.4	PE/SMT	Survival, laboratory parameters
He (2000)*	Inclusion: severe viral hepatitis according to the criteria of the 1995 national symposium Exclusion: NR	Mostly viral; alcoholic Age, sex, MELD/CTP: NR	PE, PP, DHP/SMT	Survival, laboratory parameters, HE, AEs
Hu (2005)*	Inclusion: chronic severe hepatitis complicated with multiorgan failure Exclusion: NR	NR	MARS/SMT	Survival, HE, laboratory parameters
Krisper (2005)*	Inclusion: ACLF Exclusion: NR	Mostly alcoholic; HCV Age^c^ (years): 57 Males^c^ (%): 67% MELD^b^: 35.4	MARS and Prometheus, crossover	Laboratory parameters, AEs
Laleman (2006)*	Inclusion: 18–75 years; histologically proven alcoholic cirrhosis with superposed alcoholic hepatitis; portal hypertension with associated hyperdynamic circulation and ACLF (persistent deterioration in liver function despite treatment of the precipitating event and elevated bilirubin > 12 mg%) Exclusion: extrahepatic cholestasis; coma of nonhepatic origin; active gastrointestinal bleeding in the past 5 days; comorbidities associated with poor outcome (acute necrotizing pancreatitis, neoplasia, severe cardiopulmonary disease, oxygen-dependent or steroid-dependent COPD); ongoing infection; HRS type I	Alcoholic Age^a^ (years): 54.5/43.2/55.8 Males (%): 83.3/66.7/50 MELD^a^: 22.7/29.7/24.3	MARS/Prometheus /SMT	Laboratory parameters, AEs
Meijers (2012)*	Inclusion: ≥ 18 years, compensated chronic liver disease; developed intrahepatic cholestasis (TBIL > 5 mg/dl); at least one of the following complications within 4–8 weeks after a potential identifiable acute superposed hepatic insult: (a) a progressive hyperbilirubinemia ≥ 50% increase of TBIL > 20 mg/dl, (b) HE grade ≥ II, (c) de novo development of ascites, and/or (d) HRS Exclusion: extrahepatic cholestasis; severe hypocalcemia (Ca2+ < 0.9 mmol·l^−1^); acidosis (pH < 7.25)	Mostly alcoholic; HCV, NASH, and others Age^c^ (years): 54.6 Males (%): NR MELD^a,c^: 32.1	MARS ± citrate, crossover	Laboratory parameters, AEs
Wilkinson (1998)*	Inclusion: decompensated chronic liver disease and grade III–IV encephalopathy Exclusion: NR	Alcoholic, HCV, HBV, autoimmune, unknown Age^a^ (years): 58.3/42.7 Males (%): 60/100 MELD/CTP: NR	BioLogic-DT/SMT	Physiologic and neurologic improvement, AEs
You (2011)*	Inclusion: ACLF defined by the Chinese Medical Association’s definition (2006) Exclusion: NR	Viral (?) Age^a^ (years): 42.7/43.5 Males (%): 100/83 MELD^a^: 23/24.1	HBALSS/PE	Survival, AEs, laboratory parameters

Articles included in the quantitative and qualitative synthesis (indicated by *) are listed here

*TBIL* total bilirubin, *HRS* hepatorenal syndrome, *HE* hepatic encephalopathy, *PLT* platelet, *INR* international normalized ratio, *DIC* disseminated intravascular coagulation, *RRT* renal replacement therapy, *HCC* hepatocellular carcinoma, *MAP* mean arterial pressure, *HIV* human immunodeficiency virus, *NASH* non-alcoholic steatohepatitis, *MELD* Model for end-stage liver disease, *MARS* molecular adsorbent and recirculating system, *SMT* standard medical therapy, *AEs* adverse events, *ACLF* acute-on-chronic liver failure, *ELAD* extracorporeal liver assist device, *CTP* Child–Turcotte–Pugh, *NR* not reported, *SOFA* sequential organ failure assessment, *HBV* hepatitis B virus, *PE* plasma exchange, *ARDS* adult respiratory distress syndrome, *SAH* severe alcoholic hepatitis, *AST* aspartate aminotransferase, *ALT* alanine aminotrasferase, *NAFLD* nonalcoholic fatty liver disease, *PP* plasma perfusion, *DHP* direct hemoperfusion, *HCV* hepatitis C virus, *COPD* chronic obstructive pulmonary disease

^a^Mean values

^b^Median values

^c^All patients

^d^Only reported in the intervention group

### Synthesis

#### Survival

Survival was reported in most of the included studies, with greatly varying follow-up lengths. Data synthesis was feasible in four cases: 1-month (28–31 days) and 3-month (84–91 days) data were pooled for overall and transplant-free survival. The summary of the findings for these four outcomes is presented in Table 2.

**Table 2:** Summary of findings

Summary of findings							Quality of evidence
Intervention^1^ (Studies^2^)	Rank	Study event rates (%)		Risk ratio (95% CrI)	Anticipated absolute effects		Overall certainty of evidence
		With standard medical therapy^3^	With extracorporeal liver support devices^4^		Risk with standard medical therapy	Risk difference with extracorporeal liver support devices	
3-month overall survival (follow-up: range 84 days to 91 days)							
PE (2 RCTs)	1	334/569 (58.7%)	136/244 (55.7%)	RR 0.74 (0.60 to 0.94)	59 per 100	15 fewer per 100 (from 23 to 4 fewer)	⨁◯◯◯ Very low
MARS (2 RCTs)	2		12/17 (70.6%)	RR 0.78 (0.38 to 1.40)		13 fewer per 100 (from 36 fewer to 23 more)	⨁◯◯◯ Very low
Prometheus (1 RCT)	3		46/77 (59.7%)	RR 0.97 (0.68 to 1.40)		2 fewer per 100 (from 19 fewer to 23 more)	⨁◯◯◯ Very low
ELAD (4 RCTs)	4		78/213 (36.6%)	RR 0.99 (0.76 to 1.30)		1 fewer per 100 (from 14 fewer to 18 more)	⨁◯◯◯ Very low
BioLogic-DT (1 RCT)	5		5/5 (100.0%)	RR 1.00 (0.55 to 2.10)		0 fewer per 100 (from 26 fewer to 65 more)	⨁◯◯◯ Very low
1 month overall survival (follow-up: range 28 days to 31 days)							
PE (1 RCT)	1	122/359 (34.0%)	19/104 (18.3%)	RR 0.51 (0.12 to 2.40)	34 per 100	17 fewer per 100 (from 30 fewer to 48 more)	⨁⨁⨁◯ Moderate
MARS (3 RCTs)	2		109/113 (96.5%)	RR 0.60 (0.15 to 1.30)		14 fewer per 100 (from 29 fewer to 10 more)	⨁◯◯◯ Very low
MARS + PE (indirect)	3		7/60 (11.7%)	RR 0.60 (0.07 to 3.20)		14 fewer per 100 (from 32 fewer to 75 more)	⨁◯◯◯ Very low
Prometheus (1 RCT)	4		29/77 (37.7%)	RR 1.00 (0.25 to 4.30)		0 fewer per 100 (from 25 fewer to 100 more)	⨁◯◯◯ Very low
BioLogic-DT (1 RCT)	6		6/10 (60.0%)	RR 1.10 (0.24 to 5.40)		3 more per 100 (from 26 fewer to 100 more)	⨁◯◯◯ Very low
ELAD (3 RCTs)	7		26/117 (22.2%)	RR 1.40 (0.56 to 3.60)		14 more per 100 (from 15 fewer to 88 more)	⨁◯◯◯ Very low
3-month transplant-free survival (follow-up: range 84 days to 91 days)							
PE (1 RCT)	1	189/396 (47.7%)	42/104 (40.4%)	RR 0.77 (0.51 to 1.10)	41 per 100	11 fewer per 100 (from 23 fewer to 5 more)	⨁⨁⨁◯ Moderate
Prometheus (1 RCT)	2		52/77 (67.5%)	RR 0.96 (0.67 to 1.40)		2 fewer per 100 (from 16 fewer to 19 more)	⨁◯◯◯ Very low
ELAD (4 RCTs)	4		76/217 (35.0%)	RR 1.00 (0.78 to 1.40)		0 fewer per 100 (from 11 fewer to 19 more)	⨁◯◯◯ VERY LOW
MARS (1 RCT)	5		7/8 (87.5%)	RR 1.10 (0.61 to 2.10)		5 more per 100 (from 19 fewer to 53 more)	⨁◯◯◯ Very low
1-month transplant-free survival (follow-up: range 28 days to 31 days)							
ELAD (2 RCTs)	1	109/264 (41.3%)	14/43 (32.6%)	RR 0.47 (0.13 to 1.20)	41 per 100	22 fewer per 100 (from 36 fewer to 8 more)	⨁◯◯◯ Very low
PE (1 RCT)	2		47/104 (45.2%)	RR 0.52 (0.21 to 1.20)		20 fewer per 100 (from 33 fewer to 8 more)	⨁⨁⨁◯ Moderate
MARS (3 RCTs)	3		60/122 (49.2%)	RR 0.96 (0.50 to 1.50)		2 fewer per 100 (from 21 fewer to 21 more)	⨁◯◯◯ Very low

Significant results are highlighted in italic

*CrI* credible interval, *PE* plasma exchange, *RCT* randomized controlled trial, *RR* risk ratio, *MARS* molecular adsorbent and recirculating system, *ELAD* extracorporeal liver assist device

^1^Intervention compared to SMT as reference comparator

^2^Number of studies included in the direct comparison

^3^Data from all studies

^4^Data from studies included in the direct comparison

**Fig. 2:**
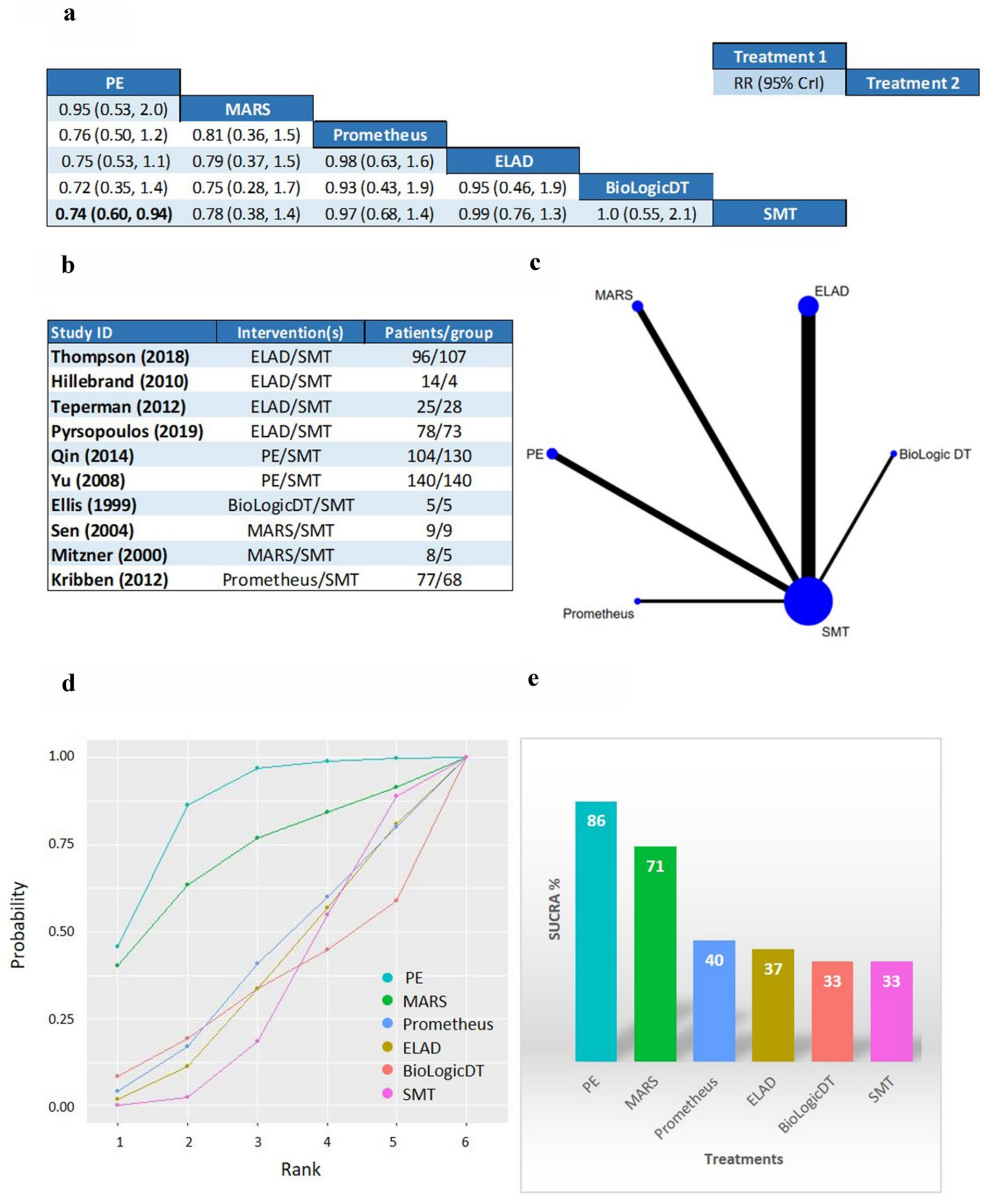
**b** Studies included in the analysis for 3-month overall survival (OS). **c** Geometry of the network: the nodes represent the number of studies and the thickness of the lines corresponds to the number of direct comparisons. **a** League table: The league table contains the risk ratios (RR) and credible intervals (CrI) for every possible comparison of the interventions. Events were defined as death during the follow-up period (84–91 days). Significant results are highlighted in bold. **d** Cumulative ranking curves: On the x axis the cumulative probability of the treatment being in the first n rank is shown, while the y axis shows the ranks. **e** Surface under the cumulative ranking curves: The surface under the cumulative ranking curve (SUCRA) is a numeric presentation of the overall ranking and presents a single number associated with each treatment. SUCRA values range from 0 to 100%. The higher the SUCRA value, and the closer to 100%, the higher the likelihood that a therapy is in the top rank or one of the top ranks. The height of each bar corresponds to the SUCRA value of the respective treatment

**Fig. 3:**
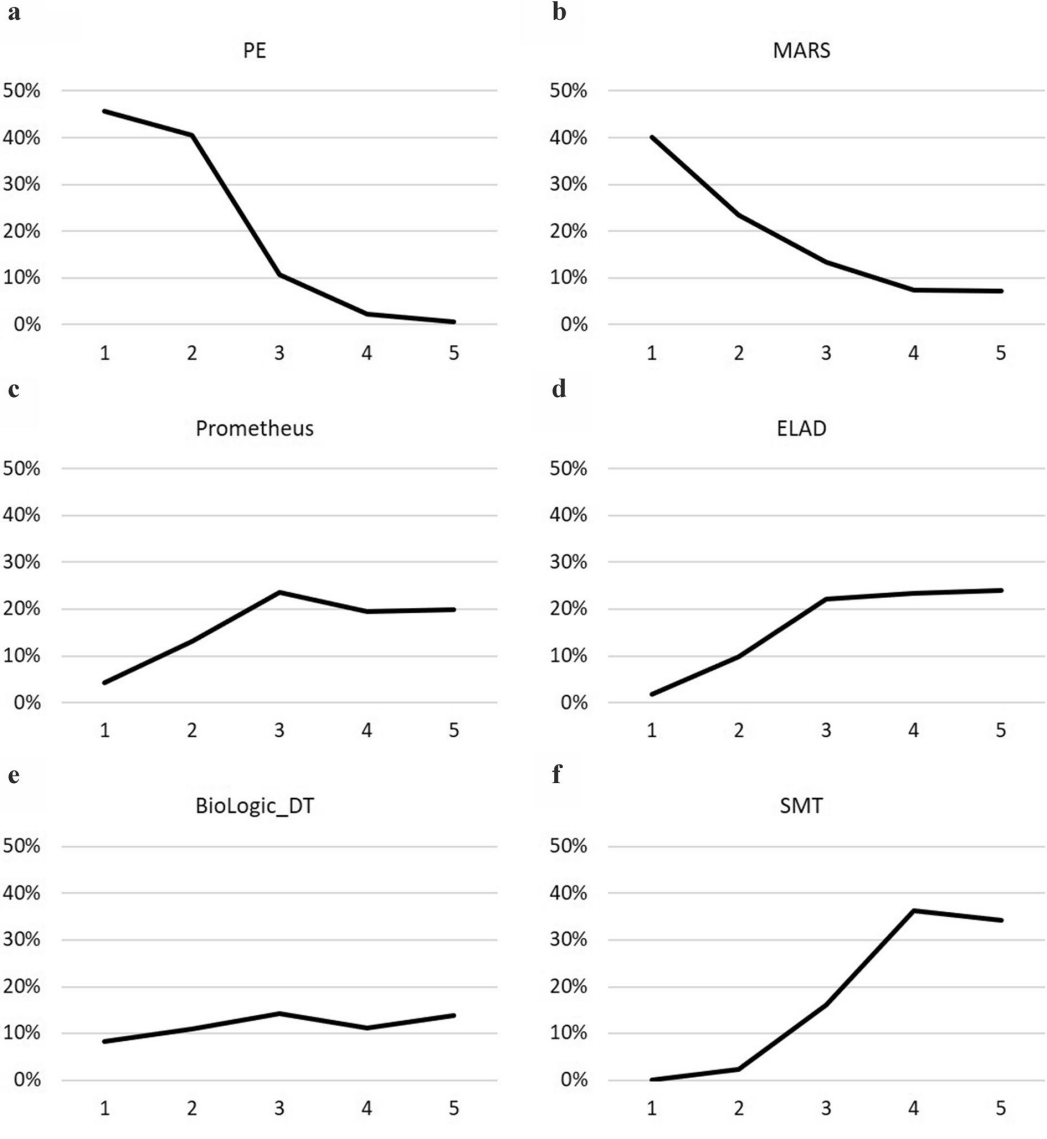
Rankograms for 3-month overall survival: Rankograms show the probability (x axis) of the respective treatment achieving certain ranks (y axis). **a** Plasma exchange, **b** molecular adsorbent and recirculating system, **c** Prometheus, **d** extracorporeal liver assist device, **e** BioLogic-DT, **f** Standard medical therapy

Plasma exchange demonstrated a statistically significant survival benefit compared to SMT in the analysis for 3-month OS (RR 0.74; CrI 0.60 to 0.94), with 86% SUCRA, 46% probability of being the best, and 41% probability of being the second-best option from the six listed treatments (Figs. 2 and 3). PE also ranked first on the cumulative curves in three out of four analyses: both 1- and 3-month OS and 1-month TFS (Fig. 2, Additional file 1: Figure S3, S7). In the analysis for 1-month TFS PE ranked second after ELAD, with 76% versus 79% SUCRA values, but had a slightly higher cumulative probability of being in the first two places than ELAD (90% versus 88%) (Additional file 1: Figure S11).

MARS ranked second in both OS outcomes (Fig. 2, Additional file 1: Figure S3) with 73% SUCRA at 1 month and 71% at 3 months. Concerning TFS, MARS ranked second last and last with SUCRA values of 27% at 1 month and 33% at 3 months (Additional file 1: Figures S7, S11). Prometheus was included in both OS analyses and in 3-month TFS. Only MARS, PE, and their combination performed better than this device in the OS outcomes and it ranked second after PE for 3-month TFS. However, the SUCRA values and the probabilities for the first ranks are much lower than for PE (SUCRA: 40% for both OS and 51% for 3-month TFS, first rank probabilities 5% for 1-month OS, 4% for 3-month OS, and 13% for 3-month TFS, shown in Figs. 2, 3, Additional file 1: Figures S3, S4, S7, S8). Despite ELAD therapy, the only biological device ranked first for 1-month TFS, in the analysis for 3-month TFS, it had a SUCRA of 38%, even lower than SMT (41%). BioLogic-DT was included in the OS analyses and ranked second last in both cases. SMT had the lowest probability of being the best or second-best option in all four analyses.

Methodology-based analyses were also performed grouping the albumin-based (MARS and Prometheus) techniques, with very similar results (only the PE-SMT comparison for 3-month OS reaching statistical significance, Additional file 1: Figures S21 and S22).

Wilkinson et al. [45] provided data only for 5-day survival comparing BioLogic-DT with SMT in a small number of patients. The device seemed to be effective in bridging to transplant. Hu et al. [46] has found that MARS improved the survival of patients with chronic severe hepatitis with multiorgan failure. You et al. [47] tested the hybrid bioartificial liver supporting system (HBALSS) in 6 patients with similar mortality rate to controls. He et al. [48] tested the effects of plasma perfusion (PP), plasma exchange (PE), and direct hemoperfusion (DHP) compared with SMT and the results were reported in Chinese. A higher survival rate was reported in the intervention group (68.75% vs 46.67%) for the whole study population. Extracted data for mortality in the ACLF subgroup by Alshamsi et al. did not show a significant difference (RR 0.59, 95% CI 0.33–1.04) [19].

Long-term survival was assessed in six studies. Six-month survival was reported to be identical in both groups by Hassanein, Heemann, and Pyrsopoulos (additionally presented at a conference, together with 1-year survival) [31, 38, 42]. Duan et al. reported higher transplant-free survival in the ELAD group, maintained until the end of the 5-year follow-up [40]. On the contrary, Thompson et al. found comparable mortality in the two groups at 5 years [39]. Interestingly, Qin et al. showed that in the PE group the 5-year cumulative survival probability was significantly higher (43% vs 31% survived) and have found that treatment added about 6 months to the life expectancy of patients with HBV-associated ACLF.

### Hepatic encephalopathy and ammonia

Altogether ten studies reported the changes in mental status, but for hepatic encephalopathy (HE) different scales and definitions were used (Additional file 1: Table S2). All studies reported improvement, which was statistically significant only in five cases, all using MARS therapy.

Ten studies reported changes in blood ammonia levels (Additional file 1: Table S4). Findings are controversial for MARS. Prometheus and BioLogic-DT do not remove ammonia effectively.

### Bilirubin

Changes in total bilirubin (TBIL) were reported in twenty studies (Additional file 1: Table S3). The results were not pooled on account of different treatment doses, measurement time points, and definitions for bilirubin reduction. Hassanein et al. rightly pointed out that the time between the last treatment session and post-treatment measurements could greatly influence this outcome [38]. They showed that a single session of MARS reduced TBIL levels significantly, but this difference decreased by the end of the 5-day treatment period. MARS, PE, MARS combined with PE, Prometheus, ELAD, and HBALSS treatments significantly reduced bilirubin levels. Krisper et al. compared MARS and Prometheus in a crossover design and reported Prometheus to be more effective in the removal of conjugated and unconjugated bilirubin. BioLogic-DT does not remove bilirubin effectively.

### Bile acids

Hassanein, Heemann, and Laleman found that both MARS and Prometheus reduced bile acid levels significantly (*P* < 0.001 and *P* < 0.001, respectively) [31, 38, 49]. Krisper et al. reported that MARS and Prometheus remove individual bile acids with different clearance rates [50]. On the other hand, Meijers et al. observed no significant reduction in bile acid levels after MARS sessions.

### Creatinine and blood urea nitrogen

Changes in creatinine levels were reported in 12 cases (Additional file 1: Table S5). Findings for MARS and BioLogic-DT are controversial regarding creatinine removal from the blood, and Prometheus and plasma exchange therapy do not influence creatinine levels.

MARS, Prometheus, and BioLogic-DT were found to decrease blood urea nitrogen levels effectively.

### Cytokines

TNF-α levels were reduced after 6 hours of BioLogic-DT treatment (*P* = 0.04) as reported by Kramer et al. [32], but only small changes were observed by Ellis et al. [37]. MARS and Prometheus treatment did not reduce TNF-α levels [34, 51]. He et al. reported significant TNF-α reduction after treatment [48]. MARS did not change IL-6, IL-8, and IL-10 levels, similarly to TNF receptors 1 and 2 [34, 51]. Higher IL-8 levels were measured in the BioLogic-DT group [37]. Levels of anti-inflammatory protein IL-1 receptor antagonist were significantly elevated for days in ELAD-treated subjects [39].

### Harms

In the numbers of adverse events (AEs) and reporting protocols, an immense heterogeneity was shown; therefore, quantitative data synthesis was not carried out. All devices were evaluated to be safe, and the number of AEs was comparable to the control groups. Hassanein et al. described nine possibly treatment-related adverse events in the MARS group; however, the nature of these was not detailed [38]. Acute hemolysis developed in one patient in the ELAD group [40] and treatment was discontinued in several subjects due to adverse events not specified [39, 41, 43]. Heemann et al. compared AEs in the MARS group to patients who received dialysis and found no significant difference. Two out of the twelve patients treated with MARS had fever/sepsis possibly related to the catheter [31].

Adverse events were reported in all but four papers in general. The most frequent complications were bleeding at the site of the catheter, clotting in the apparatus, and thrombocytopenia. Hypotension was reported in patients treated with PE and Prometheus [33, 49].

### Risk of bias assessment and quality of evidence

The quality of evidence is shown in Table 2 (see Additional file 1: Table S1 for more detail). Quality of evidence was moderate for PE in the analysis of OS at 1 month and both TFS outcomes. All other results were of very low certainty. The results of the risk of bias assessment conducted separately for OS and TFS are shown in Additional file 1: Figures S13 and S14. Overall risk of bias was low in 50% of the studies included in the OS analyses. 33% carried moderate and 22% high risk of bias. For TFS, 22% of studies carried low, 22% moderate, and 46% high risk of bias.

## Discussion

Extracorporeal liver support therapies have been and will remain of fundamental interest in the management of ACLF [52]. However, their benefits have been debated for long. Therefore, we conducted the first network meta-analysis focusing on patients with ACLF, assessing overall and transplant-free survival at 1 and 3 months. The analyses for OS yielded similar results, with PE ranking first and MARS second on the cumulative ranking curves in both cases. From all comparisons, only plasma exchange was associated with a statistically significant improvement, when compared to SMT in the analysis of 3-month overall survival, but with very low certainty of evidence. Other comparisons did not reach statistical significance, but SMT had very low probabilities of being the best option in all analyses.

Until then, evidence on the efficacy of PE in ACLF mostly originated from cohort studies. The APASL consensus guideline recommended the use of PE in ACLF for bridging to transplantation or recovery. The EASL did not find the available evidence to be sufficient for recommending the use of any liver support therapy for the treatment of ACLF. High-volume PE was found to reduce mortality and effectively remove DAMPs, TNF-α, and IL-6 in ALF patients in an RCT [53, 54].

The role of immune dysfunction and dysregulated immune response in ACLF has recently come into focus. Both hyper-inflammation and immunosuppression play a role in acute decompensation [1, 7]. Inflammation represented by elevated inflammatory markers was previously thought to be a consequence of ongoing infection, but lately endogenous inducers were identified as underlying causes [2]. Bioartificial devices have the potential of synthetic functions and contribution to the immune response [55]. So far, only ELAD was tested in RCTs, always compared to SMT. Although ELAD did not perform well on the cumulative ranking curves, significantly higher IL-1 receptor antagonist levels were measured during ELAD therapy than in controls [39]. Based on this finding, the immunomodulatory functions of bioartificial devices should be further assessed.

Several new devices are being tested in animal models of liver failure, including both artificial and bioartificial ones [56, 57], and ongoing clinical trials are enrolling ACLF patients ([58], NCT03882346, NCT04051437). Other blood purification methods, such as CytoSorb ™ therapy, also seem promising [59, 60], but they have not yet been evaluated in a randomized setting. Nevertheless, according to a recent in vitro experimental model, CytoSorb hemoperfusion leads to an initially faster removal of cytokines, like TNF-α and IL-6, as well as more effective reduction of albumin-bound toxins, such as indirect bilirubin and bile acids, compared to MARS [61].

There are some strengths and several limitations to our study. This is the first NMA in this field using the latest recommendations from the Cochrane Collaboration for statistical analysis, risk of bias, and QE assessment. We evaluated OS and TFS separately, at 1 and 3 months. We did not pool in-hospital, short-term, and long-term survival data. Studies enrolling patients with hepatorenal syndrome were not excluded with the aim of including cases with poorer prognosis. This new methodology enabled the comparison and ranking of different devices and highlighted the need for international consensus on the definition of ACLF and further trials testing already existing and new devices.

The absence of loops in all of the created networks limits statistical analysis in Bayesian networks and results in wider credible intervals. Transitivity could not be directly tested, but we think that the differences between the study populations do not violate the assumption of transitivity. The analyses included relatively few studies, some of them only enrolling less than 10 subjects per group, raising concerns about the beta-type error. Most importantly, due to the different definitions of ACLF used (Table 1), patient characteristics can differ significantly among studies, resulting in a highly heterogeneous population in our study. Eligibility criteria and the ratio of viral and alcoholic etiology differs in the included studies, but all patients were diagnosed with ACLF. Differences in the study populations may explain some of the controversial results of RCTs included in this meta-analysis. Also, in some of the included studies mortality was not a primary endpoint and was reported additionally; therefore, bias arises. The recruitment period for the included trials ranges from March 1997 until February 2015, which could impose chronological bias. Variance in SMT and treatment dose also could have influenced outcomes [62]. Due to the differences in treatment dose, cut-offs and reporting protocols, data on HE, laboratory parameters, and AEs could not be analyzed quantitatively.

## Conclusion

### Implication for practice

Plasma exchange seems to have the most beneficial effect at present, but liver support devices in general had higher probabilities for the first two ranks than SMT. Choosing the best option remains in the hands of the attending physician.

### Implication for research

International consensus is needed to standardize the definition of ACLF. Further RCTs targeting carefully selected subgroups of the ACLF population, using already existing and new therapeutic methods are needed to produce high-quality evidence for guideline development.

## Supplementary Information

**Additional file 1.** Information collected form each study, additional information used. **Figure S1.** Geometry of the network and included studies for the analysis of 1-month overall survival. **Figure S2.** League table of 1-month overall survival. **Figure S3.** Cumulative ranking curves and SUCRA values for 1-month overall survival. **Figure S4.** Rankograms for 1-month overall survival. **Figure S5.** Geometry of the network and included studies for the analysis of 3-month transplant-free survival. **Figure S6.** League table of 3-month transplant-free survival. **Figure S7.** Ranking of treatments for 3-month transplant-free survival. **Figure S8.** Rankograms for 3-month transplant-free survival. **Figure S9.** Geometry of the network and included studies for the analysis of 1-month transplant-free survival. **Figure S10.** League table of 1-month transplant-free survival. **Figure S11.** Ranking of treatments for 1-month transplant-free survival. **Figure S12.** Rankograms for 1-month transplant-free survival. **Figure S13.** Risk of bias assessment for overall survival. **Figure S14.** Risk of bias assessment for transplant-free survival. **Table S1.** Quality of evidence. **Table S2.** Assessment of hepatic encephalopathy in the included studies. **Table S3.** Assessment of bilirubin reduction in the included studies. **Table S4.** Assessment of ammonia reduction in the included studies. **Table S5.** Assessment of creatinine reduction in the included studies. **Figure S15.** Forrest plots for 3-month overall survival. **Figure S16.** Forrest plots for 1-month overall survival. **Figure S17.** Forrest plots for 3-month transplant-free survival. **Figure S18.** Forrest plots for 1-month transplant-free survival. **Figure S19.** Funnel plot and Egger’s test for 3-month overall survival. **Figure S20.** Funnel plot and Egger’s test for 1-month overall survival. **Figure S21.** Cummulative ranking curves and SUCRA for methodology-based evaluation. **Figure S22.** Methodology-based evaluation league tables.

## Data Availability

The dataset(s) supporting the conclusions of this article is(are) included within the article (and its additional file(s)).

## Abbreviations

ACLF: Acute-on-chronic liver failure
APASL: Asian Pacific Association for the Study of the Liver
AE: Adverse event
CrI: Credible interval
DAMP: Damage-associated molecular pattern
DHP: Direct hemoperfusion
EASL: European Association for the Study of the Liver
ELAD: Extracorporeal liver assist device
HBALSS: Hybrid bioartificial liver support system
HBV: Hepatitis B virus
IL: Interleukin
MARS: Molecular adsorbent recirculating system
NMA: Network meta-analysis
OS: Overall survival
PAMP: Pathogen-associated molecular patterns
PE: Plasma exchange
PP: Plasma perfusion
RCT: Randomized controlled trial
RR: Risk ratio
SMT: Standard medical therapy
SUCRA: Surface under the cumulative ranking curve
QE: Quality of evidence
TBIL: Total bilirubin
TNF-α: Tumor necrosis factor alpha
TFS: Transplant-free survival
