# Supplementary material for: Uncertainty in the impact of liver support systems in acute-on-chronic liver failure: a systematic review and network meta-analysis
Source: Ann Intensive Care. 2021 Jan 18;11:10. doi: 10.1186/s13613-020-00795-0 (PMC7813174; doi:10.1186/s13613-020-00795-0)
Supplement: Supplementary file 1 — Additional file 1. Information collected form each study, additional information used. Figure S1. Geometry of the network and included studies for the analysis of 1-month overall survival. Figure S2. League table of 1-month overall survival. Figure S3. Cumulative ranking curves and SUCRA values for 1-month overall survival. Figure S4. Rankograms for 1-month overall survival. Figure S5. Geometry of the network and included studies for the analysis of 3-month transplant-free survival. Figure S6. League table of 3-month transplant-free survival. Figure S7. Ranking of treatments for 3-month transplant-free survival. Figure S8. Rankograms for 3-month transplant-free survival. Figure S9. Geometry of the network and included studies for the analysis of 1-month transplant-free survival. Figure S10. League table of 1-month transplant-free survival. Figure S11. Ranking of treatments for 1-month transplant-free survival. Figure S12. Rankograms for 1-month transplant-free survival. Figure S13. Risk of bias assessment for overall survival. Figure S14. Risk of bias assessment for transplant-free survival. Table S1. Quality of evidence. Table S2. Assessment of hepatic encephalopathy in the included studies. Table S3. Assessment of bilirubin reduction in the included studies. Table S4. Assessment of ammonia reduction in the included studies. Table S5. Assessment of creatinine reduction in the included studies. Figure S15. Forrest plots for 3-month overall survival. Figure S16. Forrest plots for 1-month overall survival. Figure S17. Forrest plots for 3-month transplant-free survival. Figure S18. Forrest plots for 1-month transplant-free survival. Figure S19. Funnel plot and Egger’s test for 3-month overall survival. Figure S20. Funnel plot and Egger’s test for 1-month overall survival. Figure S21. Cummulative ranking curves and SUCRA for methodology-based evaluation. Figure S22. Methodology-based evaluation league tables. [file 13613_2020_795_MOESM1_ESM.docx]

**Additional material**

**Uncertainty in the impact of liver support systems in acute-on-chronic liver failure - a systematic review and network meta-analysis**

*Klementina Ocskay^1^, Anna Kanjo^1,2^, Noémi Gede^1,3^, Zsolt Szakács^1^, Gabriella Pár^4^, Bálint Erőss^1^, Jan Stange^5^, Steffen Mitzner^5^, Péter Hegyi^1,6,7^, Zsolt Molnár^1,8^*

***Information collected from each study***

First author, year of publication, number of centers, countries of data collection, number of patients randomized, number of patients per groups and loss of follow-up per group, chosen analysis strategy (ITT, PP, mITT), time of recruitment, length of follow-up, inclusion and exclusion criteria, etiology of the underlying hepatic disease, intervention, comparison, baseline characteristics, characteristics of treatments (duration, number of sessions, anticoagulation, renal replacement therapy), transplantation, outcomes. If survival was reported additionally, data was also collected.

***Additional information used***

Corresponding authors of included studies with available email adress were contacted for addition information on survival. Steffen Mitzner (Mitzner et al, 2000) kindly provided data for 3-month overall survival.

Data for 3-month transplant free survival (TFS) for the study by Pyrsopoulos et al. was taken from a presentation by Nikolaos T. Pyrsopoulos, entitled VTL-308 A Randomized, Open-label, Multicenter, Controlled, Pivotal Study to Assess Safety and Efficacy of ELAD® in Subjects with Alcohol-Induced Liver Decompensation (AILD).

**
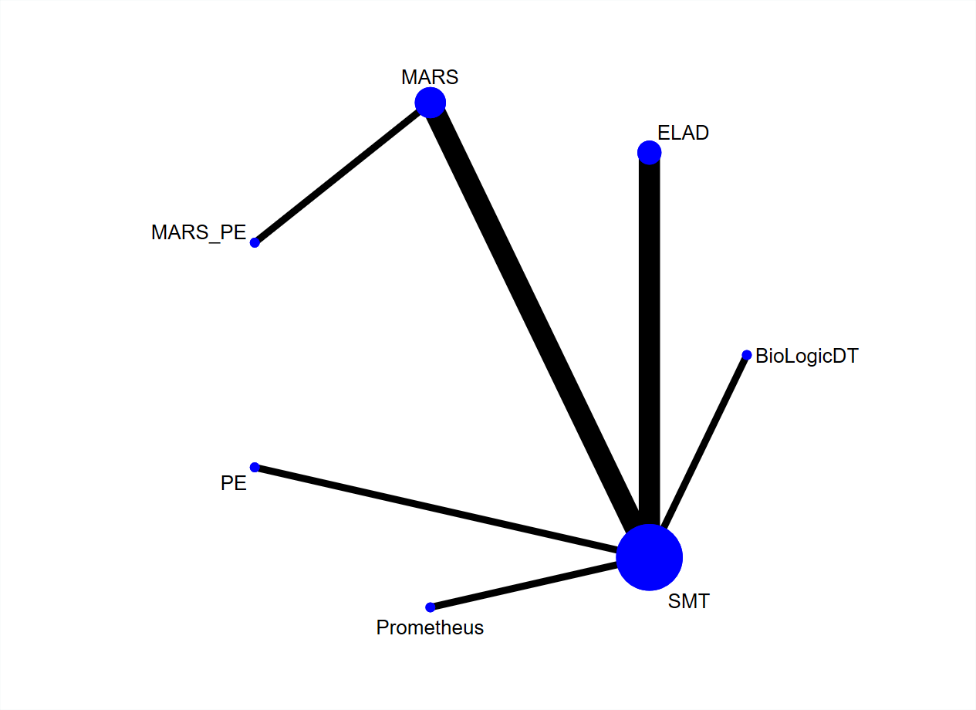
*Figure S1 Geometry of the network and included studies for the analysis of 1-month overall survival***

**B.**

**A.**

**A.** Characteristics of studies included in analysis **B.** Geometry of the network: the nodes represent the number of studies and the thickness of the lines corresponds to the number of direct comparisons. Abbreviations: ELAD: Extracorporeal Liver Assist Device; MARS: Molecular Adsorbent Recirculating System; PE: plasma exchange; SMT: standard medical therapy

***Figure S2 League table of 1-month overall survival***

******

The league table contains the risk ratios (RR) and credible intervals (CrI) for every possible comparison of the interventions. Events were defined as death during the follow-up period (28-31 days). Abbreviations: ELAD: Extracorporeal Liver Assist Device; MARS: Molecular Adsorbent Recirculating System; PE: plasma exchange; SMT: standard medical therapy

***Figure S3 Cumulative ranking curves and SUCRA values for 1-month overall survival***


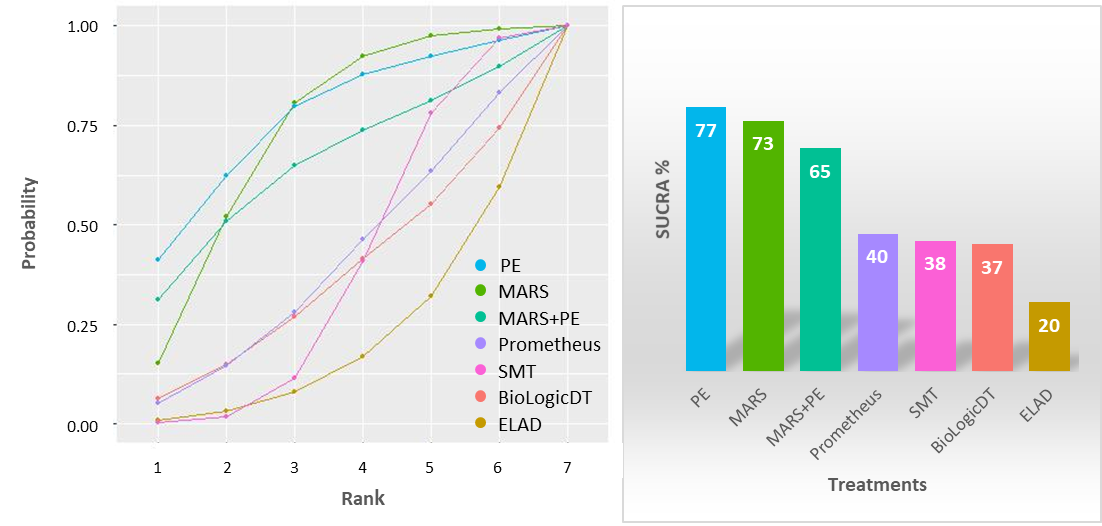


**A.**

**B.**

**A.** Cumulative ranking curves **B.** Surface under the cumulative ranking curves (SUCRA%)

A. On the *x* axis the cumulative probability of the treatment being in the first *n* rank is shown, while the *y* axis shows the ranks B. The surface under the cumulative ranking curve (SUCRA) is a numeric presentation of the overall ranking and presents a single number associated with each treatment. SUCRA values range from 0 to 100%. The higher the SUCRA value, and the closer to 100%, the higher the likelihood that a therapy is in the top rank or one of the top ranks; the closer to 0 the SUCRA value, the more likely that a therapy is in the bottom rank, or one of the bottom ranks (Mbuagbaw, 2017). The height of each bar corresponds to the SUCRA value of the respective treatment. Abbreviations: ELAD: Extracorporeal Liver Assist Device; MARS: Molecular Adsorbent Recirculating System; PE: plasma exchange; SMT: standard medical therapy

***Figure S4 Rankograms for 1-month overall survival***


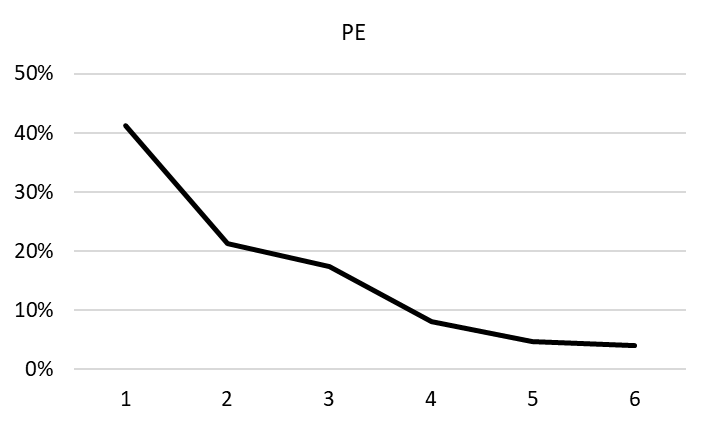

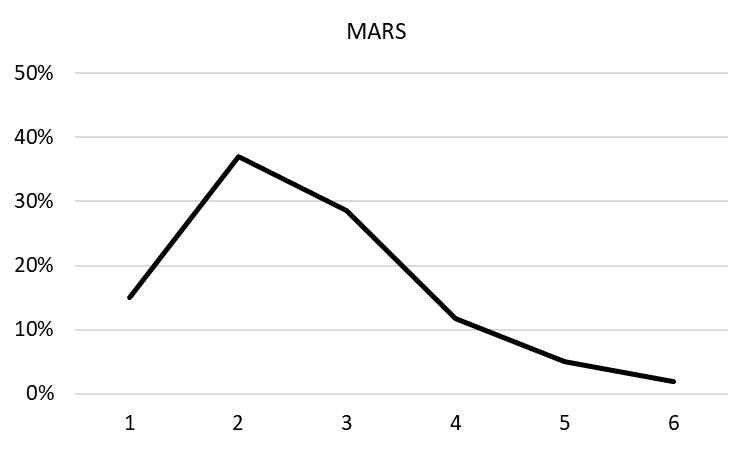


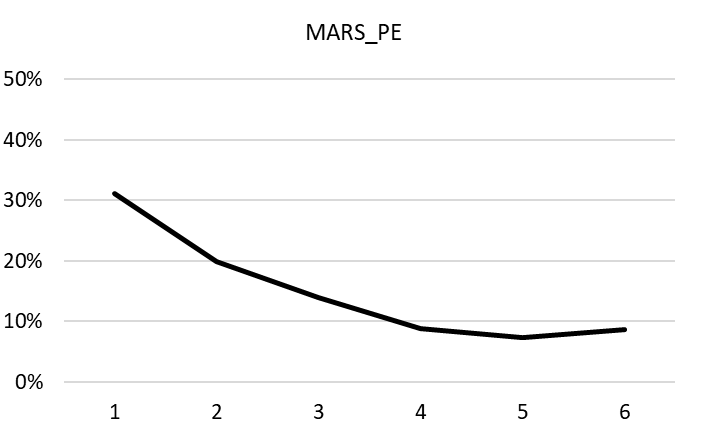

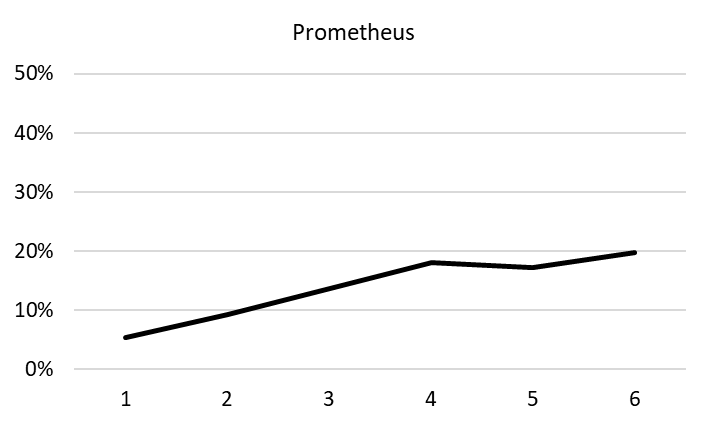


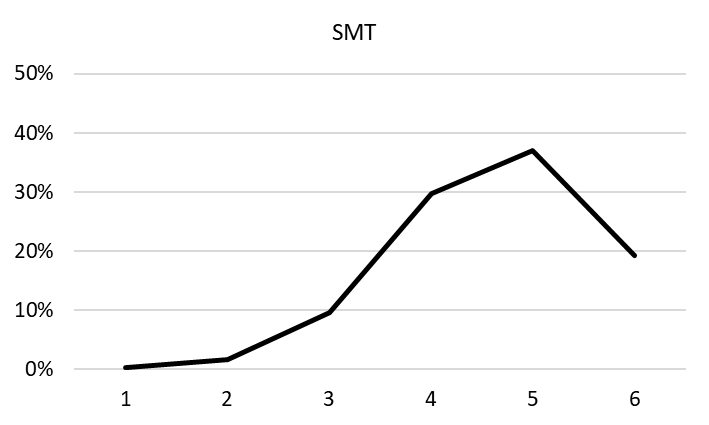

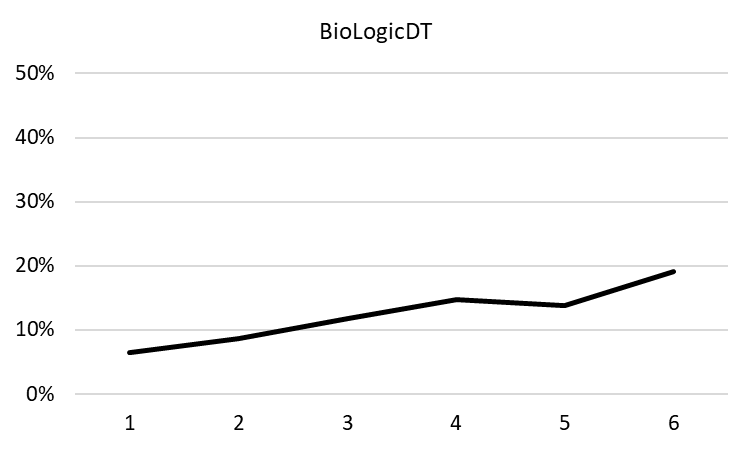


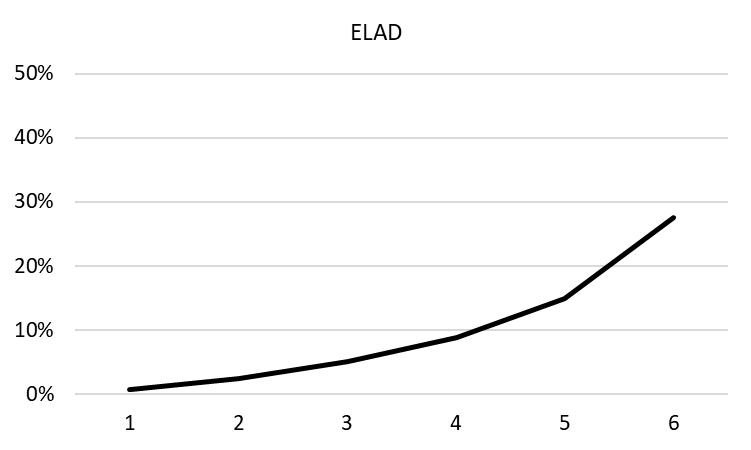


Rankograms show the probability (x axis) of the respective treatment achieving certain ranks (y axis).

***
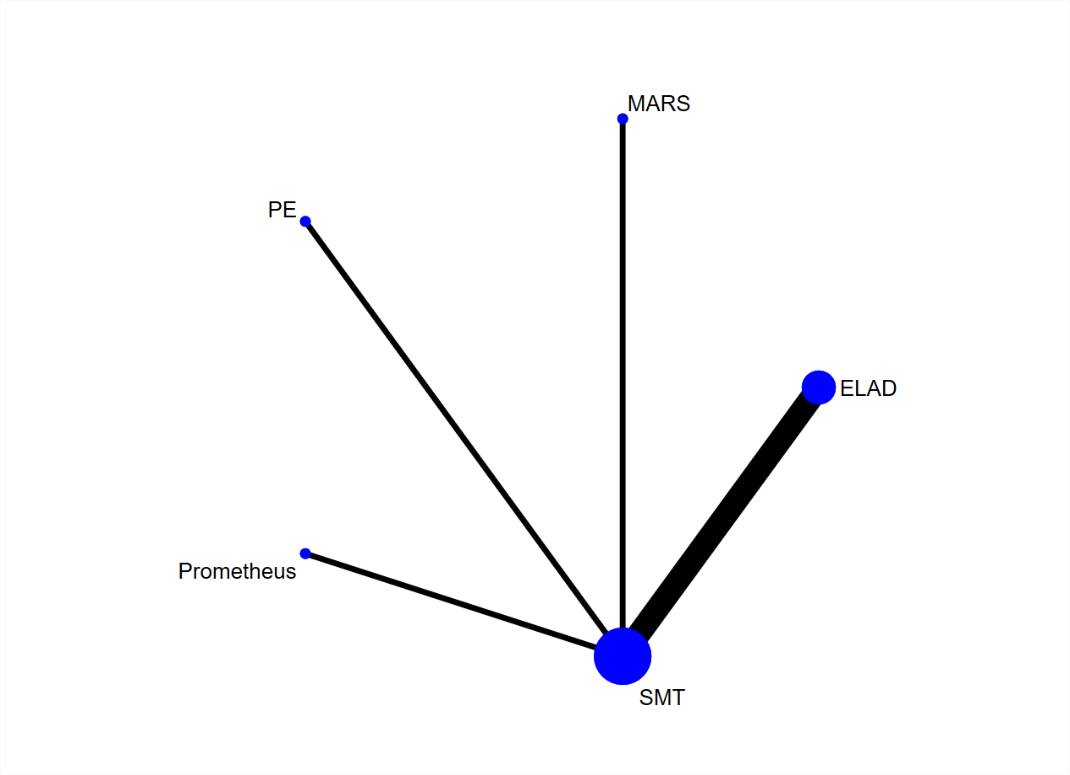
 Figure S5 Geometry of the network and included studies for the analysis of 3-month transplant-free survival***

**B.**

**A.**

**A.** Characteristics of studies included in analysis **B.** Geometry of the network: the nodes represent the number of studies and the thickness of the lines corresponds to the number of direct comparisons. Abbreviations: ELAD: Extracorporeal Liver Assist Device; MARS: Molecular Adsorbent Recirculating System; PE: plasma exchange; SMT: standard medical therapy

***Figure S6 League table of 3-month transplant-free survival***

The league table contains the risk ratios (RR) and credible intervals (CrI) for every possible comparison of the interventions. Events were defined as death or liver transplant during the follow-up period (84-91 days). Significant results are highlighted in bold. Abbreviations: ELAD: Extracorporeal Liver Assist Device; MARS: Molecular Adsorbent Recirculating System; PE: plasma exchange; SMT: standard medical therapy

***Figure S7 Ranking of treatments for 3-month transplant-free survival***


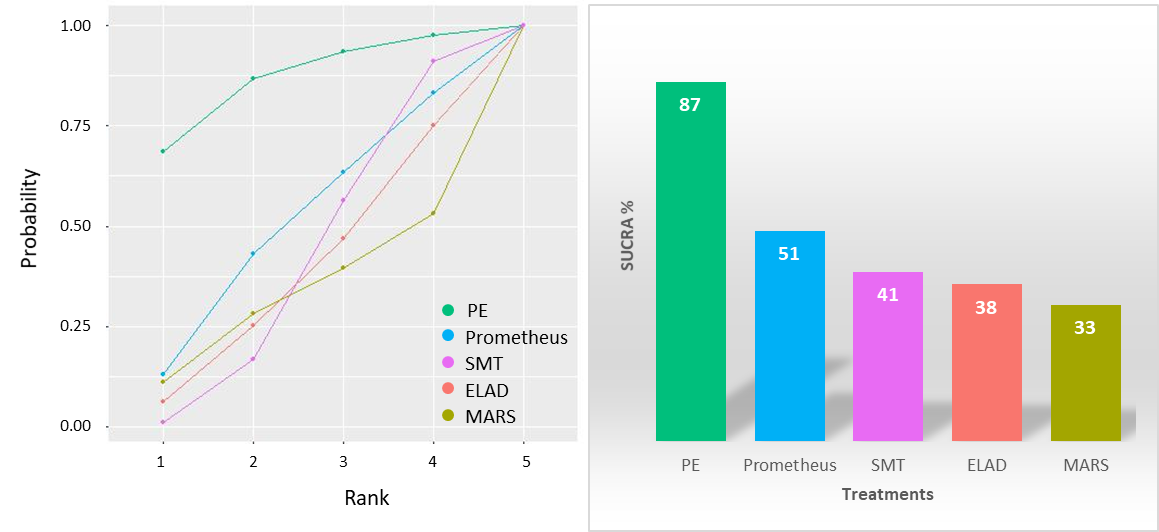


**A.**

**B.**

**A.** Cumulative ranking curves **B.** Surface under the cumulative ranking curves (SUCRA%)
A. On the *x* axis the cumulative probability of the treatment being in the first *n* rank is shown, while the *y* axis shows the ranks B. The surface under the cumulative ranking curve (SUCRA) is a numeric presentation of the overall ranking and presents a single number associated with each treatment. SUCRA values range from 0 to 100%. The higher the SUCRA value, and the closer to 100%, the higher the likelihood that a therapy is in the top rank or one of the top ranks; the closer to 0 the SUCRA value, the more likely that a therapy is in the bottom rank, or one of the bottom ranks (Mbuagbaw, 2017). The height of each bar corresponds to the SUCRA value of the respective treatment. Abbreviations: ELAD: Extracorporeal Liver Assist Device; MARS: Molecular Adsorbent Recirculating System; PE: plasma exchange; SMT: standard medical therapy

***Figure S8 Rankograms for 3-month transplant-free survival***

**
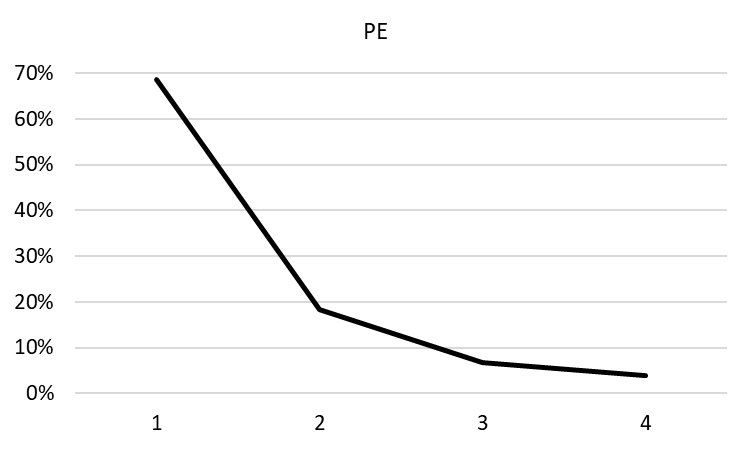
**
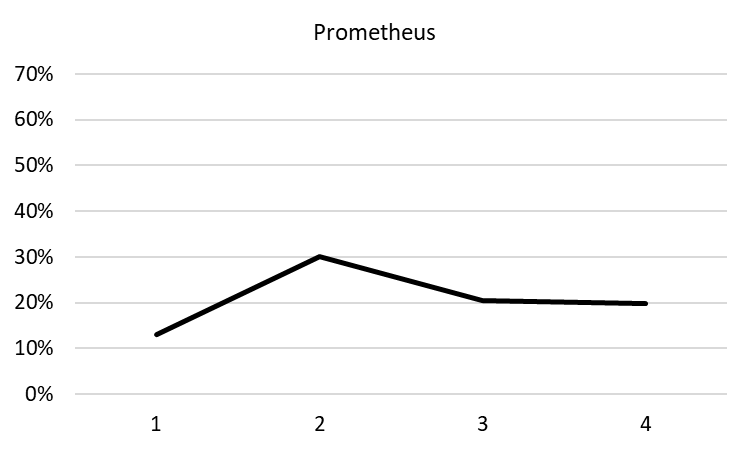


***
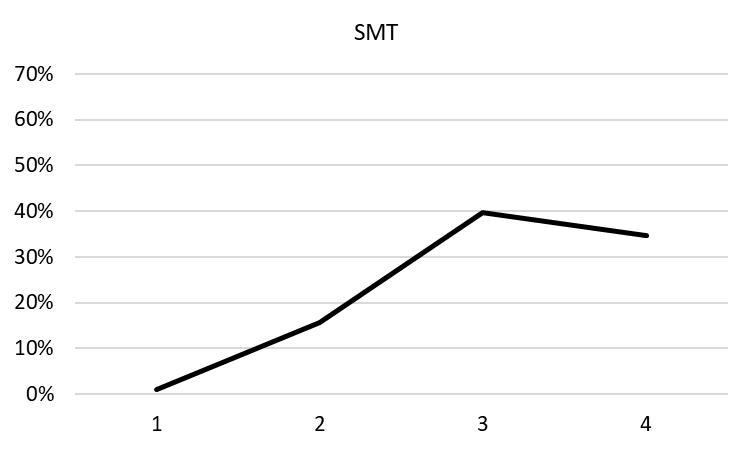
*
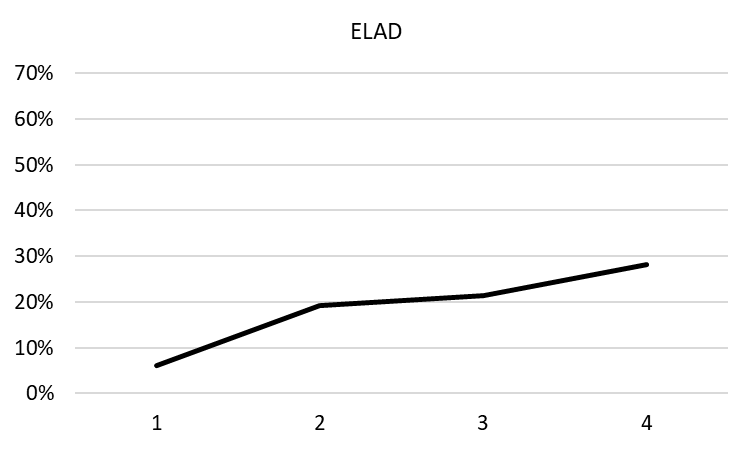
**

***
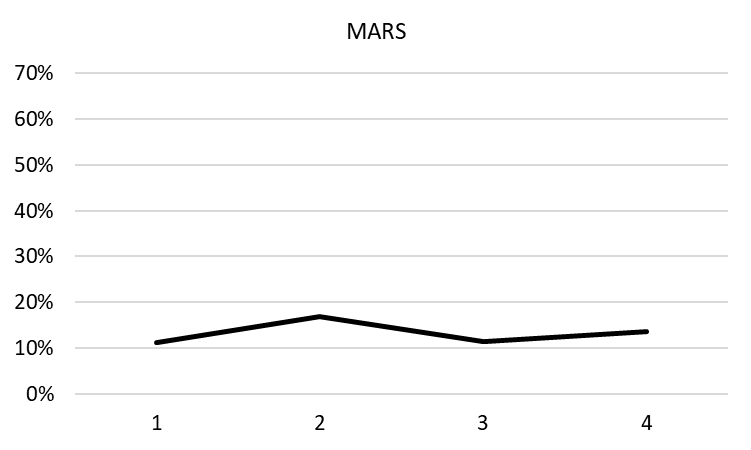
***

Rankograms show the probability (x axis) of the respective treatment achieving certain ranks (y axis).

**
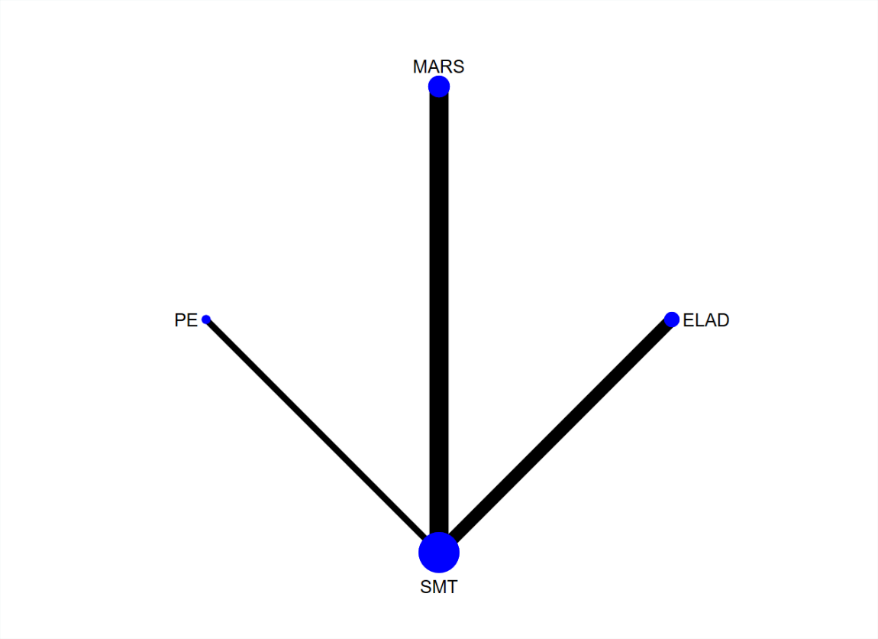
*Figure S9 Geometry of the network and included studies for the analysis of 1-month transplant-free survival***

**B.**

**A.**

**A.** Characteristics of studies included in analysis **B.** Geometry of the network: the nodes represent the number of studies and the thickness of the lines corresponds to the number direct of comparisons. Abbreviations: ELAD: Extracorporeal Liver Assist Device; MARS: Molecular Adsorbent Recirculating System; PE: plasma exchange; SMT: standard medical therapy

***Figure S10 League table of 1-month transplant-free survival***

The league table contains the risk ratios (RR) and credible intervals (CrI) for every possible comparison of the interventions. Events were defined as death or liver transplant during the follow-up period (28-31 days). Significant results are highlighted in bold. Abbreviations: ELAD: Extracorporeal Liver Assist Device; MARS: Molecular Adsorbent Recirculating System; PE: plasma exchange; SMT: standard medical therapy

***Figure S11 Ranking of treatments for 1-month transplant-free survival***

***
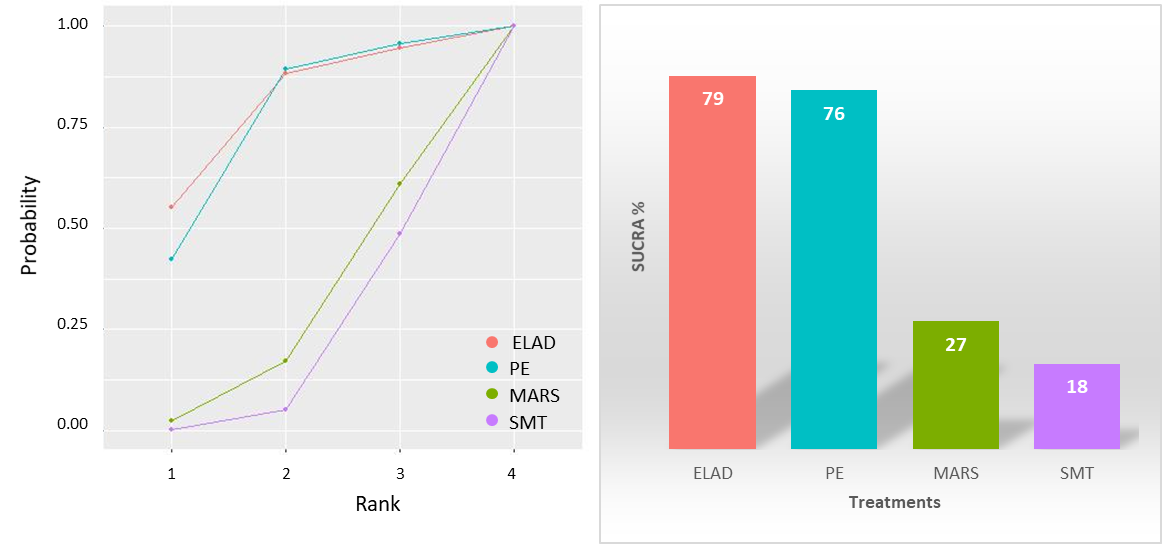
***

**A.**

**B.**

**A.** Cumulative ranking curves **B.** Bar plot of the SUCRA values of each treatment.

A. On the *x* axis the cumulative probability of the treatment being in the first *n* rank is shown, while the *y* axis shows the ranks B. The surface under the cumulative ranking curve (SUCRA) is a numeric presentation of the overall ranking and presents a single number associated with each treatment. SUCRA values range from 0 to 100%. The higher the SUCRA value, and the closer to 100%, the higher the likelihood that a therapy is in the top rank or one of the top ranks; the closer to 0 the SUCRA value, the more likely that a therapy is in the bottom rank, or one of the bottom ranks (Mbuagbaw, 2017). The height of each bar corresponds to the SUCRA value of the respective treatment. Abbreviations: ELAD: Extracorporeal Liver Assist Device; MARS: Molecular Adsorbent Recirculating System; PE: plasma exchange; SMT: standard medical therapy

***Figure S12 Rankograms for 1-month transplant-free survival***


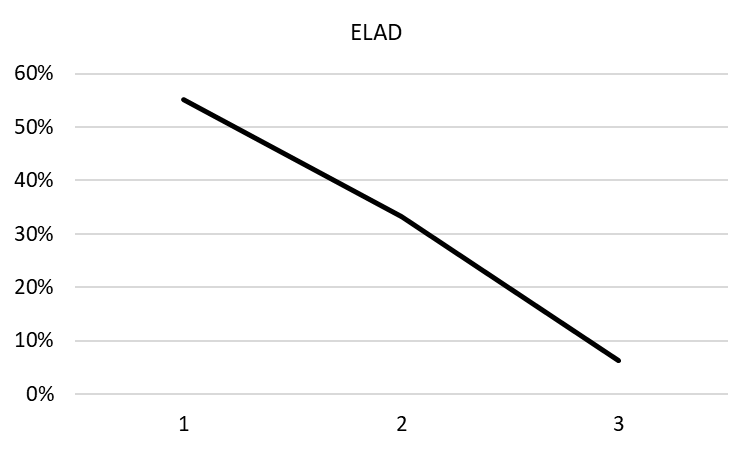

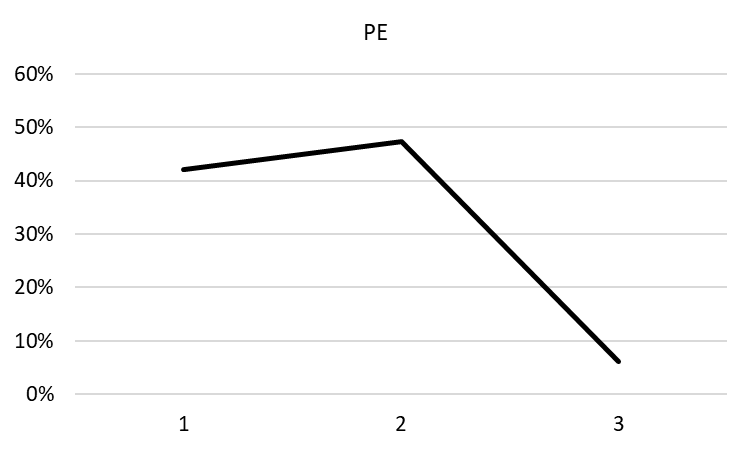


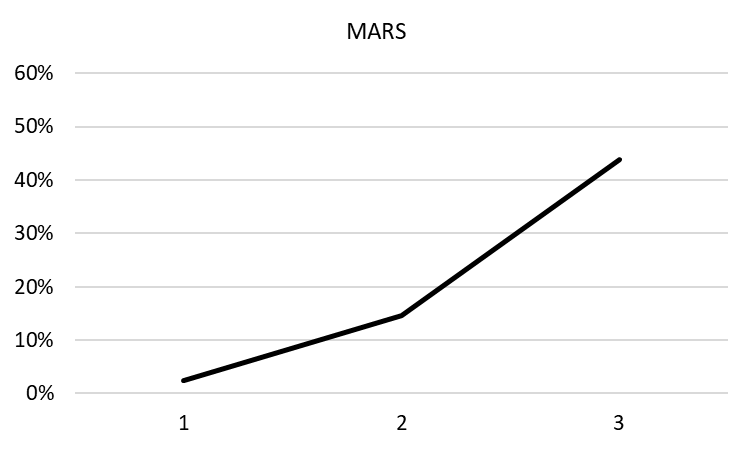

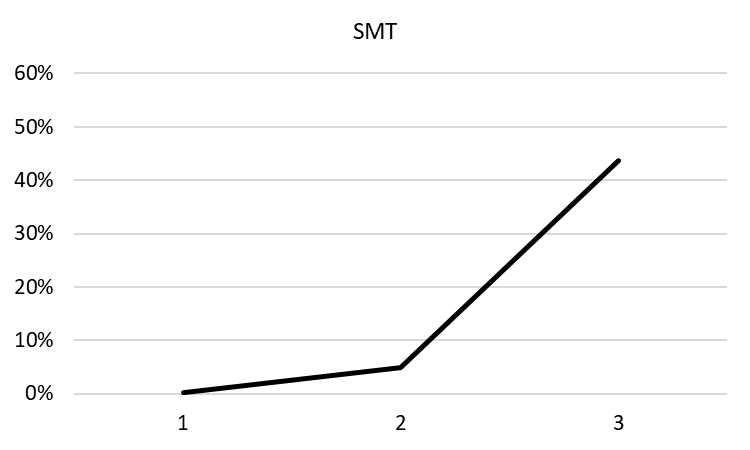


Rankograms show the probability (x axis) of the respective treatment achieving certain ranks (y axis).

**Figure S13 Risk of bias assessment for overall survival**

**A.**

**B.**

**
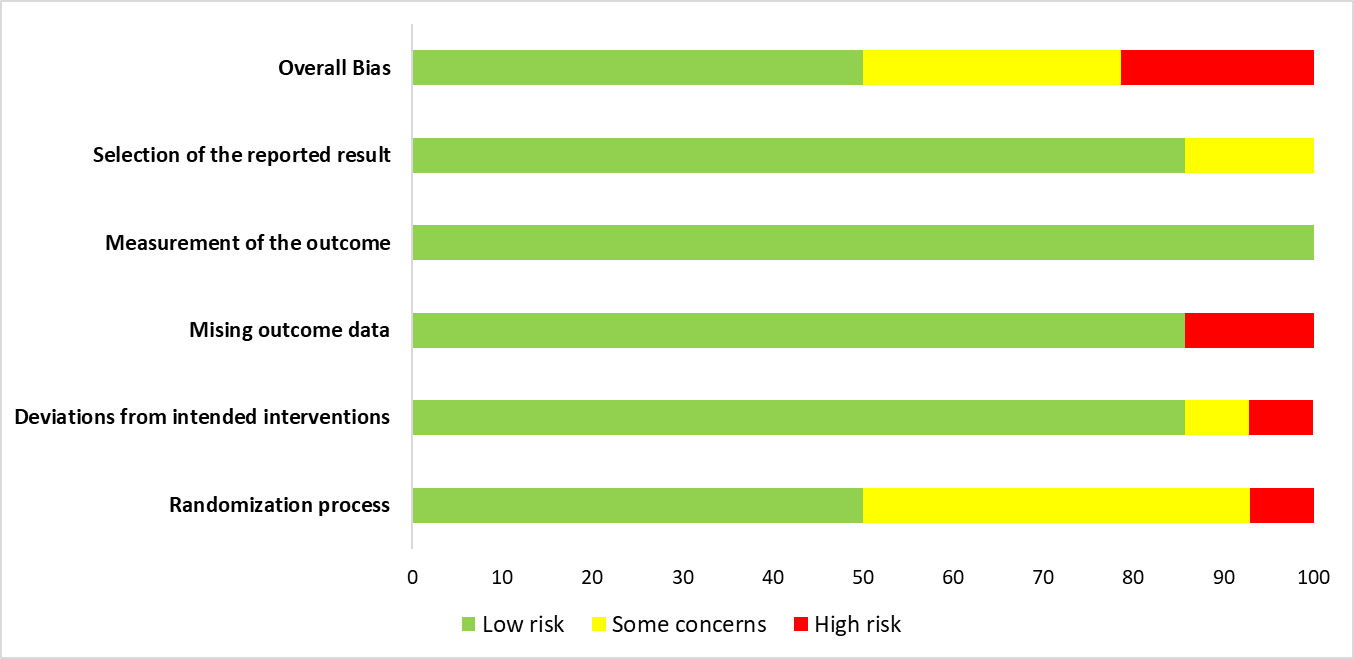
**

**A.** Risk of bias for individual studies **B.** Risk of bias across studies

Version 2 of the Cochrane risk-of-bias tool for randomized trials (RoB 2) was used for assessment. The ‘Selection of the reported result’ domain was judged to carry low risk of bias because we only analysed raw data in most cases. Abbreviations: ELAD: Extracorporeal Liver Assist Device; MARS: Molecular Adsorbent Recirculating System; PE: plasma exchange; SMT: standard medical therapy

**Figure S14 Risk of bias assessment for transplant-free survival**

**A.**

**B.**

***
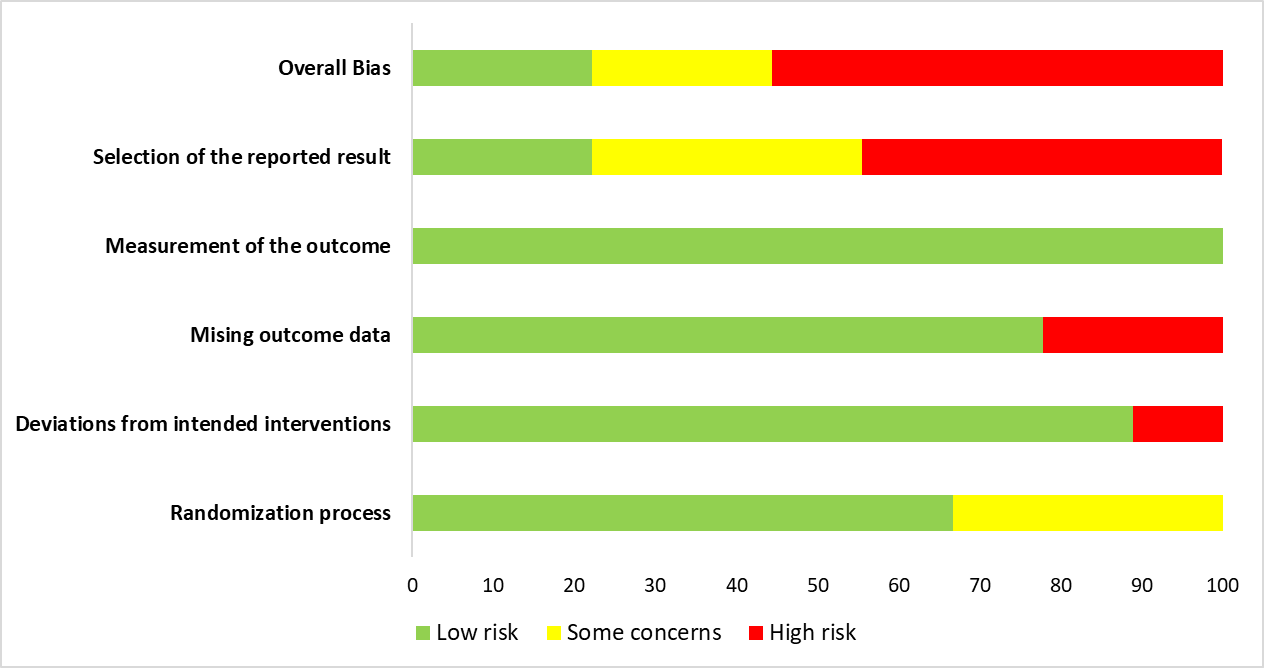
***

**A.** Risk of bias for individual studies **B.** Risk of bias across studies

Version 2 of the Cochrane risk-of-bias tool for randomized trials (RoB 2) was used for assessment. Abbreviations: ELAD: Extracorporeal Liver Assist Device; MARS: Molecular Adsorbent Recirculating System; PE: plasma exchange; SMT: standard medical therapy

**Table S1 Quality of evidence**

| **3-month overall survival (follow up: range 84 days to 91 days)** | | | | | | |
| --- | --- | --- | --- | --- | --- | --- |
| **Certainty assessment** | | | | | | |
| **Intervention^1^ (Studies^2^)** | **Risk of bias^3^** | **Inconsistency^4^** | **Indirectness** | **Imprecision^5^** | **Publication bias^6^** | **Overall certainty of evidence** |
|  |  |  |  |  |  |  |
| PE (2 RCTs) | very serious ^a^ | not serious | serious ^d^ | not serious ^e^ | none | ⨁◯◯◯ VERY LOW |
| MARS (2 RCTs) | not serious | not serious | serious ^b^ | very serious ^c,g^ | none | ⨁◯◯◯ VERY LOW |
| Prometheus (1 RCT) | very serious ^a^ | not serious | not serious | serious ^c^ | none | ⨁◯◯◯ VERY LOW |
| ELAD (4 RCTs) | serious ^f^ | not serious | serious ^h,i^ | very serious ^c,g^ | none | ⨁◯◯◯ VERY LOW |
| 574 (1 RCT) | serious ^f^ | not serious | not serious | very serious ^c,g^ | none | ⨁◯◯◯ VERY LOW |

| **Certainty assessment** | | | | | | |
| --- | --- | --- | --- | --- | --- | --- |
| **Intervention^1^ (Studies^2^)** | **Risk of bias^3^** | **Inconsistency^4^** | **Indirectness** | **Imprecision^5^** | **Publication bias^6^** | **Overall certainty of evidence** |
|  |  |  |  |  |  |  |
| PE (1 RCT) | not serious ^e^ | not assessed | serious ^f^ | not serious ^g^ | none | ⨁⨁⨁◯ MODERATE |
| MARS (3 RCTs) | very serious ^a^ | not assessed | serious ^c^ | very serious ^d^ | none | ⨁◯◯◯ VERY LOW |
| MARS+PE (indirect) | serious ^h^ | not assessed | serious ^f^ | serious ^d^ | none | ⨁◯◯◯ VERY LOW |
| Prometheus (1 RCT) | very serious ^a^ | not assessed | not serious | serious ^d^ | none | ⨁◯◯◯ VERY LOW |
| BioLogicDT (1 RCT) | serious ^h^ | not assessed | not serious | very serious ^d,i^ | none | ⨁◯◯◯ VERY LOW |
| ELAD (3 RCTs) | not serious | not assessed | serious ^j^ | very serious ^d,i^ | none | ⨁◯◯◯ VERY LOW |

| **Certainty assessment** | | | | | | |
| --- | --- | --- | --- | --- | --- | --- |
| **Intervention^1^ (Studies^2^)** | **Risk of bias^3^** | **Inconsistency^4^** | **Indirectness** | **Imprecision^5^** | **Publication bias^6^** | **Overall certainty of evidence** |
|  |  |  |  |  |  |  |
| PE (1 RCT) | not serious | not assessed | serious ^d^ | not serious | not assessed | ⨁⨁⨁◯ MODERATE |
| Prometheus (1 RCT) | very serious ^a^ | not assessed | not serious | serious ^c^ | not assessed | ⨁◯◯◯ VERY LOW |
| ELAD (4 RCTs) | very serious ^a^ | not assessed | serious ^f,g^ | very serious ^c,e^ | not assessed | ⨁◯◯◯ VERY LOW |
| MARS (1 RCT) | not serious | not assessed | serious ^b^ | very serious ^c,e^ | not assessed | ⨁◯◯◯ VERY LOW |

**3-month transplant-free survival (follow up: range 84 days to 91 days)**

| **Certainty assessment** | | | | | | |
| --- | --- | --- | --- | --- | --- | --- |
| **Intervention^1^ (Studies^2^)** | **Risk of bias^3^** | **Inconsistency^4^** | **Indirectness** | **Imprecision^5^** | **Publication bias^6^** | **Overall certainty of evidence** |
|  |  |  |  |  |  |  |
| ELAD (2 RCTs) | very serious ^a^ | not assessed | very serious ^e^ | serious ^c,f^ | not assessed | ⨁◯◯◯ VERY LOW |
| PE (1 RCT) | not serious | not assessed | serious ^d^ | not serious | not assessed | ⨁⨁⨁◯ MODERATE |
| MARS (3 RCTs) | very serious ^a^ | not assessed | serious ^b^ | very serious ^c,e^ | not assessed | ⨁◯◯◯ VERY LOW |

#### Explanations

1: Intervention compared to SMT as reference comparator

2: Number of studies included in the direct comparison

3: Risk of bias (RoB) of studies included in direct comparison or in case of indirect comparison all studies on the arm were considered and in case of at least 1 study with high RoB, overall RoB was considered very serious, in case of studies with moderate risk of bias the overall RoB was considered serious.

4: Node splitting could not be performed due to the geometry of the networks, as a result inconsistency could not be tested. No automatic downgrading was performed.

5: Based on the sample size calculations of included studies, a 100 patient per study arm minimum sample size was defined.

6: Funnel plots were created and Egger’s tests were performed to assess small-study effect for OS. Publication bias were assessed at outcome level. Too few studies were included in the analyses for TFS, therefore publication bias were not assessed for these outcomes.

a. study with high risk of bias included in this comparison

b. etiologies differ across studies, one study only enrolled patients with HRS

c. estimated sample size per group was around 100 patients (Qin, Kribben), none of the studies reached this sample size

d. study only enrolled HBV patients

e. sample size is larger than the estimated minimum

f. study with moderate risk of bias included in this comparison

g. very small sample size

h. missing information on study population

i. enrolled patients differ from the general population in age and etiology

**Table S2 Assessment of hepatic encephalopathy in the included studies**

| **First author, publication year** | **Device** | **Definition/assessment of HE** | **Patient number** | **Time frame and treatment dose** | **Results** | **Improvement of HE** |
| --- | --- | --- | --- | --- | --- | --- |
| **Banares (2013)** | MARS | marked improvement of hepatic encephalopathy (decrease from grade II-IV to grade 0-I) | 179 | no time frame given; treatments on the first 4 days, followed by 3 sessions/w until sustained improvement, max. 10 sessions in 21 days | improvement of hepatic encephalopathy: OR: 0.37; 95% CI 0.12-1.09; P=0.07; | not significant |
| **Ellis (1999)** | BioLogicDT | improvement in HE grade (equivalent to West-Haven) | 10 | day 1 and 4; 6 hours of dialysis daily for 3 days | The severity of clinical encephalopathy improved over the course of 3 treatments (4 days) in 3 patients, remained unchanged in 1 and got worse in the other within the BioLogicDT treated group. There were no significant changes following a single session of sorbent dialysis. By comparison there was improvement in encephalopathy grade in 1 patient in the control group while 3 remained unchanged and one died by Day 4 | not significant |
| **Hassanein (2007)** | MARS | improvement proportion: the number of HE assessments with at least 2 grades improvement in HE from baseline divided by the total number of assessments done during the study | 70 | anytime during the 5 day study period; 6 hours daily, max. 5 treatments | MARS was more effective than the SMT group in resolving advanced stages of HE. Time to improvement was significantly shortened in the MARS group compared with the SMT group (P=0.044) in spite of the 5.25 hours’ delay in starting MARS after randomization. | significant |
| **He (2000)** | PE, PP, DHP | NR | 64 | NR | DHP improved HE | NR |
| **Heemann (2002)** | MARS | improvement in HE grade (West-Haven) | 23 | measured continously for 30 days; max. 10 treatments, max. 1 treatment per day, some days skipped | The improvement in HE grade persisted throughout the 30 days of the study for the MARS group. The signed rank statistic for pre/post-differences indicated that albumin dialysis resulted in a significant improvement of the HE score for the MARS group at 1 week, whereas HE progressed in thecontrol group significantly during weeks 2, 3, and 4. At any visit after MARS treatment, there was a significantly lower degree of HE in the MARS group compared with the control group (P for Mann-Witney U test at weeks 1, 2, 3 and 4: <0.01, <0.005, <0.01, <0.005). | significant |
| **Hu (2005)** | MARS | NR | 82 | NR | MARS therapy clinically presented in significant therapeutic effectiveness in hepatic encephalopathy or brain edema | significant  (p value not presented) |
| **Huang (2012)** | MARS±PE | Glasgow coma score (awake rate after treament) | 120 | 3 day post-treatment; daily treatments until patient wakes up | The GCS was significantly increased in both group after 3 days of treatment (both p<0.0001) Awake rate did not differ between groups (P=0.769) | significant |
| **Kramer (2001)** | BioLogicDT | clinical stage (by Conn), EEG | 20 | after 6 hours of treatment | The clinical stage of HE did not change significantly during the study period in either group. Latency of N70 peak improved in 9 of 10 treated as compared to 4 of 10 control patients (P = 0.056) and mean latency of N70 improved in treated patients only (P = 0.005). Similarly, cervico-cranial transmission time and N70 amplitude improved during treatment whereas the changes in control patients were not significant. EEG median and edge frequencies did not change significantly during treatment. | not significant |
| **Sen (2004)** | MARS | improvement in HE grade (West-Haven) | 18 | post-treatment, daily measurements for 7 days | Encephalopathy improved significantly in the MARS group over individual MARS sessions as well as over the 7-day study period, but not in the SMT group. In the MARS group only 11.1% had not shown an improvement (defined as reduction by 1 grade) by day 2, while in the SMT group 38.1% had not improved even at the end of the study period (P <.01). Two patients in the MARS group showed a subsequent worsening of encephalopathy by 1 grade, related to infection in 1 and variceal bleeding in the other. | significant |
| **Wilkinson (1998)** | BioLogicDT | neurologic status (Caronna scale) | 8 | pre- to post-treatment, dose varies | Treated patients had an increase in neurologic status (2 out of 5 improved, P=0.65), though this increase was slightly less than that found in nonrandomized studies. Control patients exhibited no change in neurologic status. | not significant |

Abbreviations: HE: hepatic encephalopathy; MARS: Molecular Adsorbent Recirculating System; PE: plasma exchange; PP: plasma perfusion; DHP: direct hemoperfusion; SMT: standard medical therapy; NR: not reported

**Table S3 Assessment of bilirubin reduction in the included studies**

| **First author, publication year** | **Device** | **Change in TBIL given as** | **Time frame and treatment dose** | **Patient number** | **Results** | **Dercrease of TBIL** |
| --- | --- | --- | --- | --- | --- | --- |
| **Banares (2013)** | MARS | percentage of change, means compared | baseline and day 4; treatments on the first 4 days, followed by 3 sessions until sustained improvement, max. 10 sessions in 21 days | 179 | The use of MARS as compared with SMT was associated with a significant reduction in serum bilirubin at day 4 (P<0.001), but these effects were no longer maintained at day 21. | significant |
| **Duan (2018)** | ELAD | mean decrease in % | end-of-treatment; minimum of 3 days, maximum of 5 days (note: concomittant therapies included plasma bilirubin absorption) | 49 | TBIL level decreased by 25% during ELAD treatment vs 37% increase in the control group (P<0.001).The overall magnitude of decrease of TBIL and direct bilirubin (DBil) from baseline in the ELAD group was 25.1% and 20.4%, respectively, compared to an increase in the control group of 36.8% and 33.7%. On day 14, the difference between the treatment groups was not statistically significant for TBIL or DBIL | significant |
| **Ellis (1999)** | BioLogicDT | medians compared | day 1 and 4; 6 hours of dialysis daily for 3 days | 10 | Over the three day study period and the subsequent day the pattern for the routine blood tests of liver function (including AST, plasma bilirubin and INR) was similar in the BioLogic-DT treated and control patients. | no change |
| **Hassanein (2007)** | MARS | single treatment effect and medians compared at baseline and end of treatment | single treatment and end of study (day 5); 6 hours daily, max. 5 treatments | 70 | The single treatment effect for the 108 MARS treatments resulted in the decrease of median values for TBIL [16.4 (0.2-54.5) to 16.1 (1.9-42.1) mg/dL (P <0.01)], but no significant reduction at baseline versus EOS (P=0.064) | significant for single treatment |
| **He (2000)** | PE, PP, DHP | means compared | not specified | 64 | treatment group vs control group (48.3 ±18.5 vs 57.2 ± 23.7 umol/L) p<0.01 | significant |
| **Heemann (2002)** | MARS | decrease in serum bilirubin (morning levels <15 mg/dL for 3 consecutive days) was the primary end point, medians compared | day after treatment; standard dose calculated | 23 | The primary end point was reached in 5 of 12 patients in the MARS group and in 2 of 12 patients (2 of 11 per protocol) in the control group. The median time to reach this end point was shorter in the MARS group. Serum bilirubin levels were decreased significantly on the consecutive day after MARS treatment, from 25 (13 to 50) mg/dL to 23 (13 to 50) mg/dL (P <0.005). | significant |
| **Huang (2012)** | MARS±PE | means compared | 3 day post-treatment; daily treatments until patient wakes up | 120 | Serum total bilirubun levels were significantly decreased in both groups (P<0.001) | significant |
| **Kramer (2001)** | BioLogicDT | medians compared | after 6 hours of treatment | 20 | No relevant changes in the levels of bilirubin (P=0.76) | no change |
| **Kribben (2012)** | Prometheus | means compared, change given | day 28; first week 5 treatments, 3+3 additional treatment on the 2nd and 3rd weeks | 145 | Treatment with Prometheus was associated with a significant reduction (P<0.001) in serum bilirubin levels compared with SMT alone. | significant |
| **Krisper (2005)** | MARS vs Prometheus (crossover) | reduction ratio | pre- and post-tretament; 6 hour treatments | 8 | Significantly higher reduction ratios for Prometheus compared to MARS were found: 37±7 vs. 28±6% for TBIL,16±16 vs. -4±12% for unconjugated bilirubin and 52±12 vs. 43±7% for conjugated bilirubin. | two modalities compared |
| **Laleman (2006)** | MARS | changes compared in % | post-treatment, day 3 and 7; 6 hour treatments 3 successive days | 18 | Both Prometheus and MARS decreased serum bilirubin levels (P < 0.005 versus SMT), the Prometheus device being more effective than MARS (P = 0.002). Re-evaluation of bilirubin levels 3 and 7 days after termination of the treatment period showed a comparable bilirubin level in the MARS-treated group at day 3 to post-treatment (P = 0.455), which increased at day 7 (P = 0.042 versus end of treatment). A similar evolution was noted in the Prometheus group (P=0.590 and P=0.046). In the SMT group, TBIL remained stable  (P = 0.396 and P =0.840). | significant |
|  | Prometheus |  |  |  |  | significant |
| **Meijers (2012)** | MARS ± citrate (crossover) | reduction ratio | pre- and post-tretament; 6 hour treatments daily, max. not defined | 10 | Between group comparison demonstrated citrate anticoagulation to significantly increase the likelihood of completed MARS treatment (Fisher’s exact test, P=0.04). This translates into higher bilirubin reduction ratios when citrate was applied (reduction ratio 0.25 vs. 0.15, P=0.02). | two modalities compared |
| **Mitzner (2000)** | MARS | means compared | post-treatment; max. 10 treatments, 1 treatment per day | 13 | A significant decrease in bilirubin levels (P<0.01) were observed in the MARS group. | significant |
| **Pyrsopoulos (2019)** | ELAD | Proportion of Subjects with Early Change in Bilirubin Level | up to day 7; tretaments on 5 successive days | 151 | P<0.05 (not reported in abstract, results from trial register) | significant |
| **Sen (2004)** | MARS | Cutoff of 20% at Study Day 7 | day 7; 4 sessions of 8 hours, over the 7-day study period | 18 | A significant improvement of serum bilirubin over 7 days was seen in the MARS group (P<0.001), but not in the SMT group. | significant |
| **Teperman (2012)** | ELAD | mean reduction from baseline, categorical analysis based on 10% threshold change from baseline | days 1-4; treatment regimen not specified | 44 | In the PP analysis, ELAD subjects but not SMT subjects had significant reductions from baseline in total bilirubin during ELAD therapy (days 1, 2, 3 and 4). Mean reduction from baseline for ELAD subjects was 20% at days 3 and 4 p<0.01) while SMT subjects had a mean increase of 4% and 8%, respectively. Categorical analysis based on 10% threshold change from baseline total bilirubin showed significant differences between ELAD and SMT subjects on days 1-4 (p<0.01). | significant |
| **Thompson (2018)** | ELAD | number of patient with >20% reduction and means compared | day 2-7; ELAD treatment was conducted continuously for 120 hours unless subjects deteriorated and became futile, withdrew consent, or subjects responded quickly after 72 hours. | 203 | ELAD treatment resulted in a significant reduction of total bilirubin compared with controls at all time points from day 2 to day 7. Significantly more ELAD subjects (56/95, 59%) reached a bilirubin reduction over 20% compared with controls (25/108, 23%) by day 7. (In one subject ELAD was discontinued due to futility (bilirubin increase >25%)). | significant |
| **Wilkinson (1998)** | BioLogicDT | mean changes presented | pre- to post-treatment, dose varies | 8 | Treatment with the BioLogic-DT System resulted in an insignificant increase in bilirubin. The upward trend in bilirubin after treatment was probably related to volume depletion of the patients (2-3 L fluid removal). | countereffective |
| **You (2011)** | HBALSS | changes illustrated in treatment group | pre- to post-treatment, after 1 week, dose varies | 6 | P<0.05 (comparison not specified) | significant |
| **Yu (2008)** | PE | means compared (pre- and post-treatment) | pre- to post-treatment, PE therapy was carried out twice every week until the patient’s condition was stable | 280 | TBIL levels were singnificantly lower than before PE treatment (P<0.05) | significant |

Abbreviations: TBIL: total bilirubin; DBIL: direct bilirubin; ELAD: extracorporeal liver assist device; MARS: Molecular Adsorbent Recirculating System; PE: plasma exchange; PP: plasma perfusion; DHP: direct hemoperfusion; HBALSS: hybrid bioartificial liver support system; SMT: standard medical therapy

**Table S4 Assessment of ammonia reduction in the included studies**

| **First author, publication year** | **Device** | **Change in ammonia given as** | **Time frame and treatment dose** | **Patient number** | **Results** | **Reduction** |
| --- | --- | --- | --- | --- | --- | --- |
| **Ellis (1999)** | BioLogicDT | means compared | day 1 and 4; 6 hours of dialysis daily for 3 days | 10 | Levels of arterial ammonia were raised in both BioLogicDT treated patients and the control group on entry to the study. The ammonia level rose slightly during the first two treatments in the BioLogic treated group and subsequently fell after 3 treatments, but this was not statistically significant. There was no such change in the control group. | not significant |
| **Hassanein (2007)** | MARS | medians compared | single treatment and end of study (day 5); 6 hours daily, max. 5 treatments | 70 | The single treatment effect for the 108 MARS treatments resulted in the decrease of serum ammonia (P <0.01); end of study P=0.001 | significant |
| **He (2000)** | PE, PP, DHP | means compared | treatment group vs controls after treatment | 64 | p<0.01 | significant |
| **Huang (2012)** | MARS±PE | means compared | 3 day post-treatment; daily treatments until patient wakes up | 120 | Blood ammonia levels were significantly decrease after 3 days of treatment in the MARS group (P<0.001) and in the PE+MARS group (P<0.0001). | significant |
| **Kramer (2001)** | BioLogicDT | medians compared | after 6 hours of treatment | 20 | Baseline ammonia was elevated in 8 treated and 6 control patients and did not change significantly in either group (P=0.58). | no change |
| **Krisper (2005)** | MARS vs Prometheus (crossover) | reduction ratio | pre- and post-tretament; 6 hour treatments | 8 | Despite significantly higher ammonia clearance for Prometheus, there was no significant difference in the ammonia reduction ratio: 40±18 vs. 35±14% for Prometheus and MARS, respectively. | two modalities compared |
| **Laleman (2006)** | MARS | means compared | post-treatment; 6 hour treatments 3 successive days | 18 | (pre- and post-treatment levels displayed) | no change |
|  | Prometheus |  |  |  |  | no change |
| **Sen (2004)** | MARS | medians compared | post-tretament, day 7; 4 sessions of 8 hours, over the 7-day study period | 18 | Plasma ammonia was elevated 4- to 5-fold and did not change significantly in either group. No difference in concentration was detected either over 7 days or over individual MARS sessions even though ammonia was detected in the dialyzate of the MARS circuit, from which it was found to be removed across the hemofilter column | no change |
| **Wilkinson (1998)** | BioLogicDT | mean change displayed | pre- to post-treatment, dose varies | 8 | Treatment with the BioLogic-DT System resulted in an insignificant increase in plasma ammonium. The upward trend in ammonium after treatment was probably related to volume depletion. A time course of ammonium in treated patients showed a large drop after the first treatment. The ammonium level then became stable. | increase |
| **You (2011)** | HBALSS | only displayed in treatment group | NR | NR | ammonia levels showed a slight increase after treatment and decreased to pre-treatment levels 3 days after therapy | NR |

Abbreviations: TBIL: total bilirubin; DBIL: direct bilirubin; ELAD: extracorporeal liver assist device; MARS: Molecular Adsorbent Recirculating System; PE: plasma exchange; PP: plasma perfusion; DHP: direct hemoperfusion; HBALSS: hybrid bioartificial liver support system; SMT: standard medical therapy; NR: not reported

**Table S5 Assessment of creatinine reduction in the included studies**

| **First author, publication year** | **Device** | **Change in creatinine given as** | **Time frame and treatment dose** | **Patient number** | **Results** | **Dercrease of creatinine** |
| --- | --- | --- | --- | --- | --- | --- |
| **Banares (2013)** | MARS | percentage of change, means compared | baseline, day 4 and 21; treatments on the first 4 days, followed by 3 sessions/w until sustained improvement, max. 10 sessions in 21 days | 179 | The use of MARS as compared with SMT was associated with a significant reduction in serum creatinine at day 4, but these effects were no longer maintained at day 21 | significant |
| **Ellis (1999)** | BioLogicDT | medians compared | day 1 and 4; 6 hours of dialysis daily for 3 days | 10 | Serum creatinine tended to be higher in the control cases | not significant |
| **Hassanein (2007)** | MARS | single treatment effect and medians compared at baseline and end of treatment | single treatment and end of study (day 5); 6 hours daily, max. 5 treatments | 70 | The single treatment effect for the 108 MARS treatments resulted in the decrease of median values for creatinine (P<0.01), end of study P=0.001 | significant |
| **He (2000)** | PE, PP, DHP | means compared | treatment group vs controls after treatment | 64 | p<0.01 | significant |
| **Heemann (2002)** | MARS | medians compared | baseline, week 2 and 4; max. 10 treatments, max. 1 treatment per day | 23 | No statistically significant differences were found for creatinine. | not significant |
| **Kramer (2001)** | BioLogicDT | medians compared | after 6 hours of treatment | 20 | P=0.79 | no change |
| **Kribben (2012)** | Prometheus | means compared, change | day 28; first week 5 treatments, 3+3 additional treatment on the 2nd and 3rd weeks | 145 | P=0.60 for change from baseline | no change |
| **Laleman (2006)** | MARS | changes compared in % | post-treatment; 6 hour treatments 3 successive days | 18 | There were no differences between the treatment groups with regard creatinine levels. | not significant |
|  | Prometheus |  |  |  |  | not significant |
| **Mitzner (2000)** | MARS | means compared | post-treatment; max. 10 treatments, 1 treatment per day | 13 | In the MARS group, the following changes were observed for serum creatinine: 3.8± 1.6 versus 2.3±1.5 mg/dL (P<0.01) | significant |
| **Sen (2004)** | MARS | medians compared | day 7; 4 sessions of 8 hours, over the 7-day study period | 18 | No significant change of renal function (serum creatinine, urine output) was observed in either group. | no change |
| **Wilkinson (1998)** | BioLogicDT | mean changes presented | pre- to post-treatment, dose varies | 8 | Treatment with the BioLogic-DT System resulted in a significant decrease in creatinine. | significant |
| **Yu (2008)** | PE | means compared (pre- and post-treatment) | pre- to post-treatment, PE therapy was carried out twice every week until the patient’s condition was stable | 280 | The levels of serum creatinine were low before PE treatment, but there was no significant difference afterwards. | no change |

Abbreviations: TBIL: total bilirubin; DBIL: direct bilirubin; ELAD: extracorporeal liver assist device; MARS: Molecular Adsorbent Recirculating System; PE: plasma exchange; PP: plasma perfusion; DHP: direct hemoperfusion; SMT: standard medical therapy

**Figure S15 Forrest plots for 3-month overall survival**


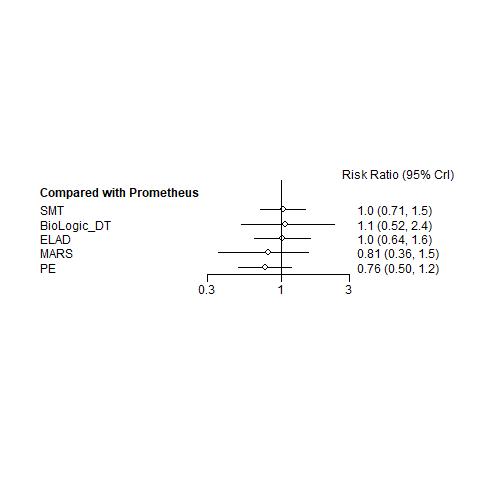

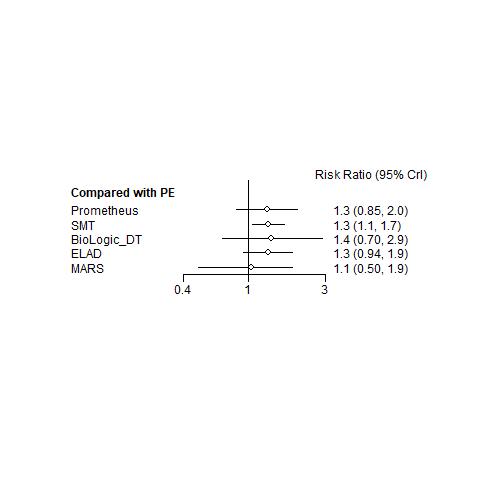

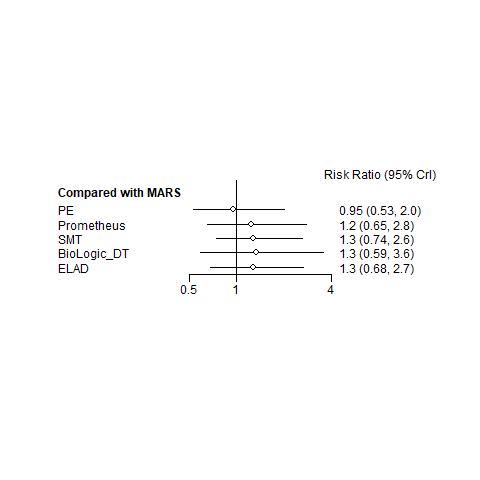

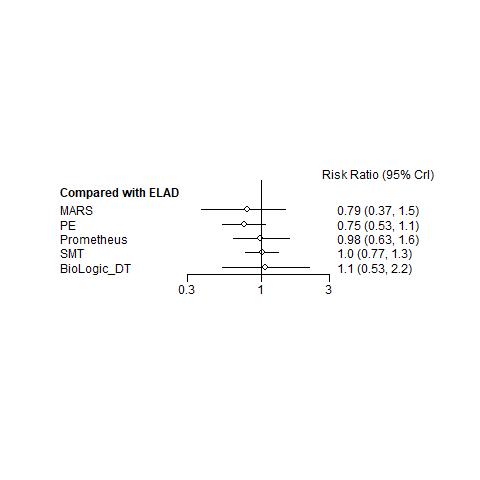

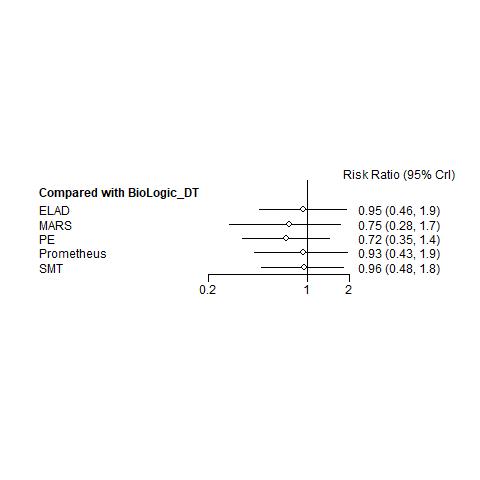

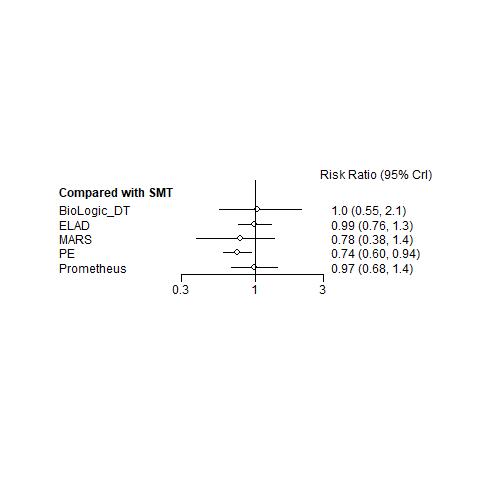


The Forrest plot shows the interventions compared to a certain comparator (listed on top). Vertical lines illustrate the credible interval (CrI) and the white ball in the midle the risk ratio (of death). If the CrI crosses the solid vertical line of no effect, the difference is not significant statistically.

**Figure S16 Forrest plots for 1-month overall survival**


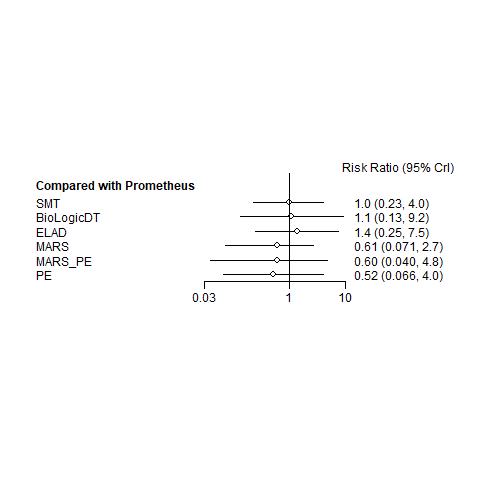

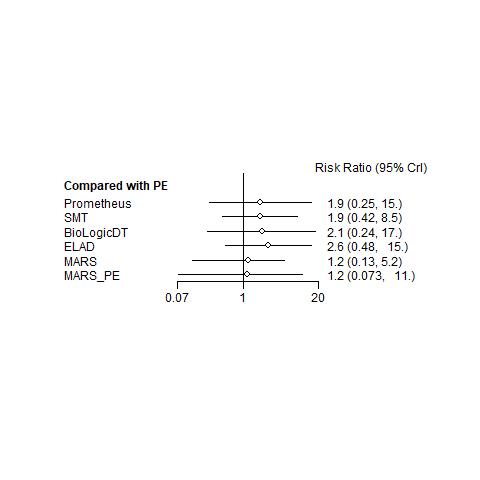

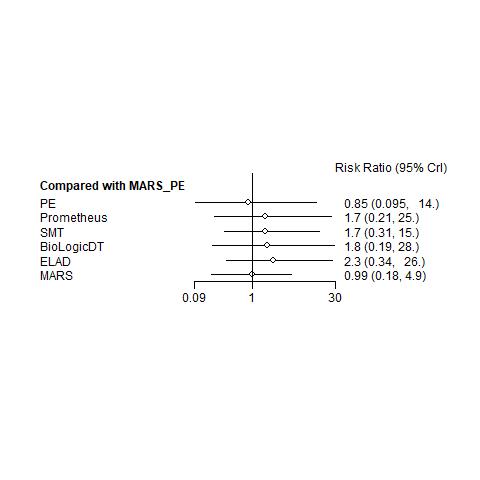

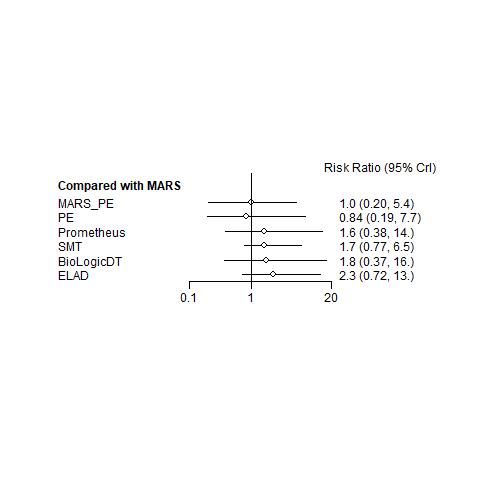

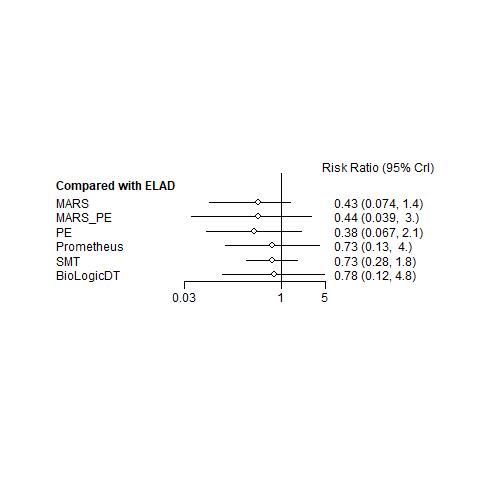


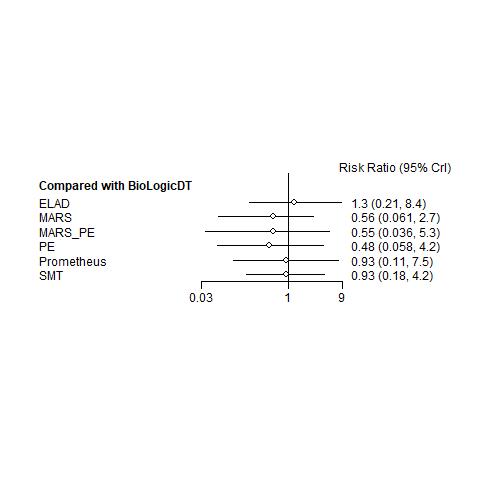

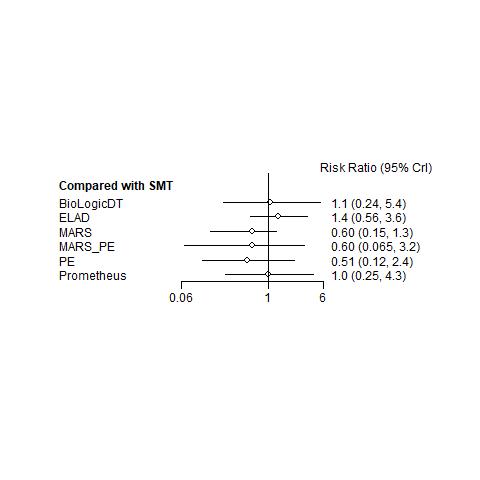


The Forrest plot shows the interventions compared to a certain comparator (listed on top). Vertical lines illustrate the credible interval (CrI) and the white ball in the midle the risk ratio (of death). If the CrI crosses the solid vertical line of no effect, the difference is not significant statistically.

**Figure S17 Forrest plots for 3-month transplant-free survival**


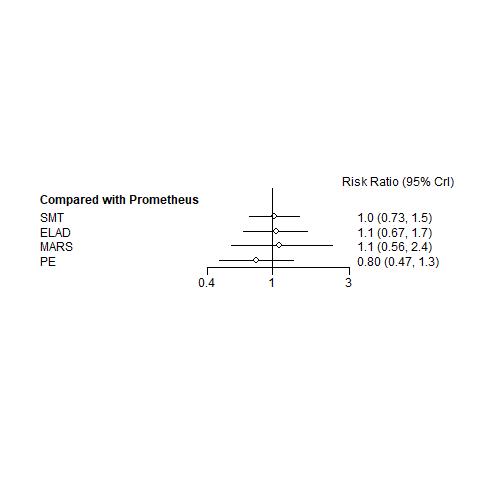

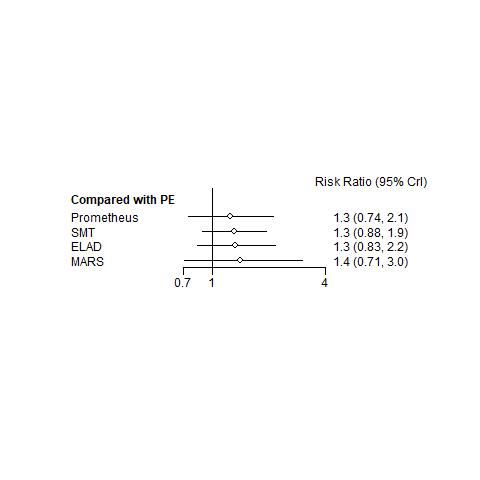

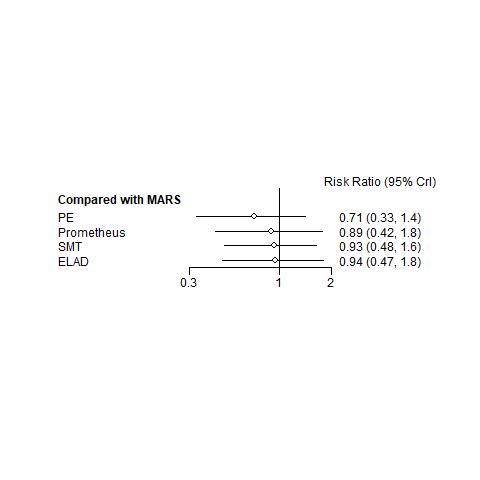

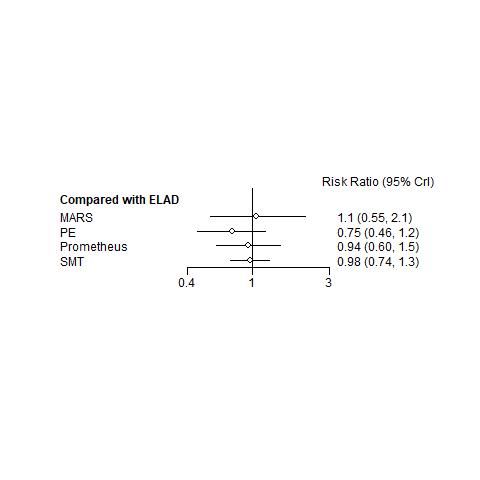

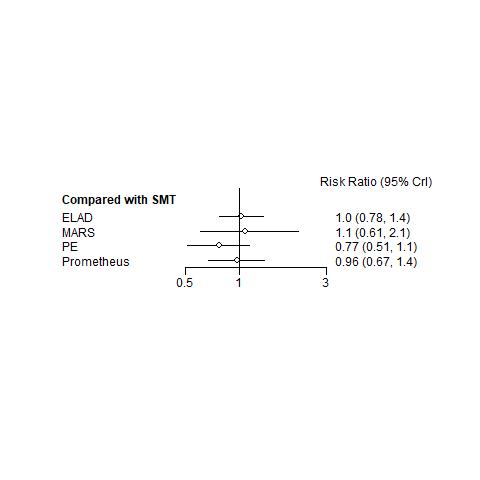


The Forrest plot shows the interventions compared to a certain comparator (listed on top). Vertical lines illustrate the credible interval (CrI) and the white ball in the midle the risk ratio (for transplant or mortality). If the CrI crosses the solid vertical line of no effect, the difference is not significant statistically.

**Figure S18 Forrest plots for 1-month transplant-free survival**
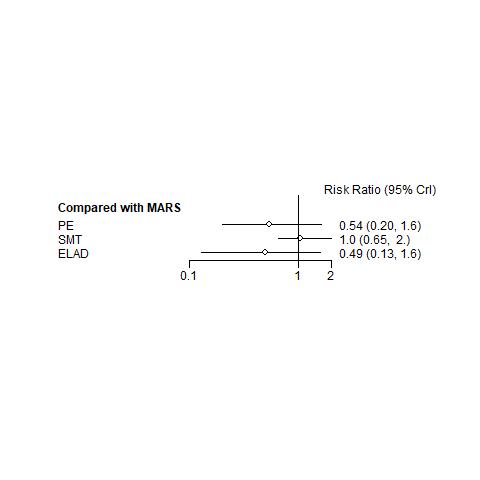

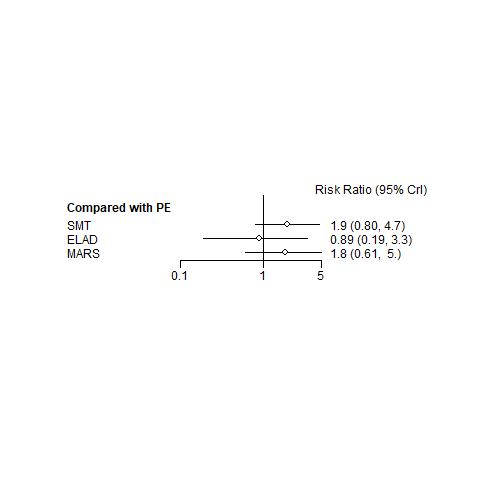


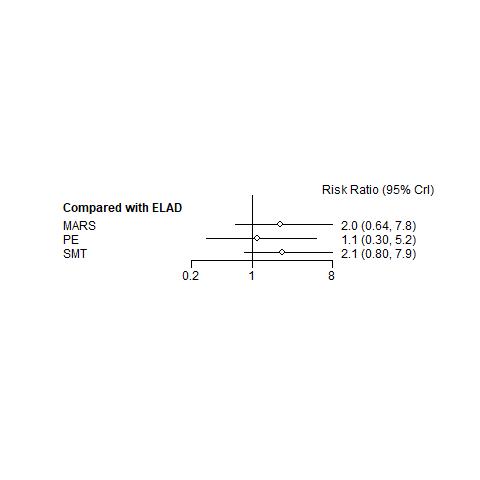

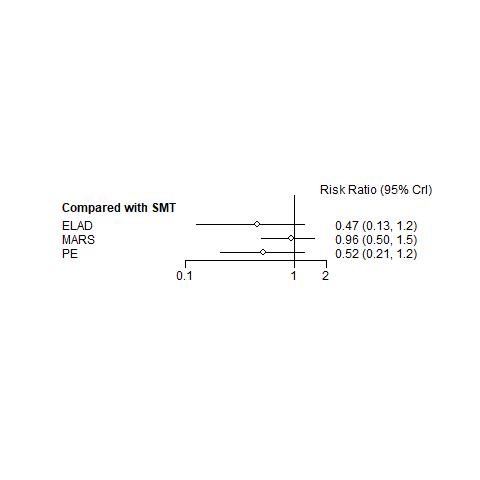


The Forrest plot shows the interventions compared to a certain comparator (listed on top). Vertical lines illustrate the credible interval (CrI) and the white ball in the midle the risk ratio (for transplant or mortality). If the CrI crosses the solid vertical line of no effect, the difference is not significant statistically.

**Figure S19 Funnel plot and Egger’s test for 3-month overall survival**


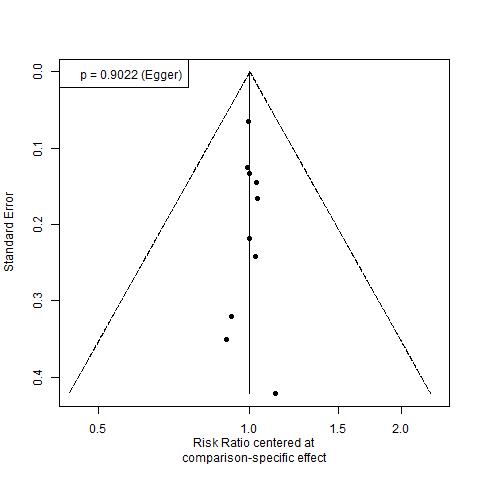


Visual assessment and the Egger’s test does not implicate publication bias.


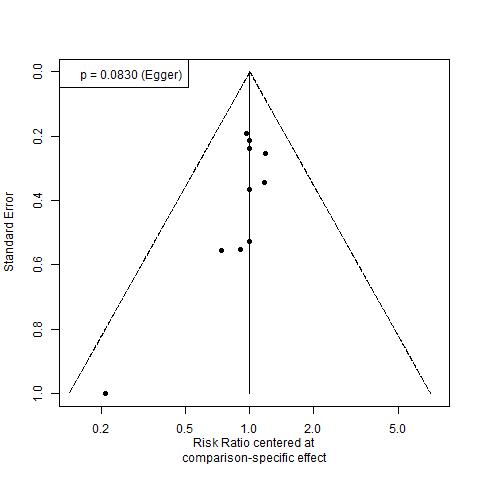
**Figure S20 Funnel plot and Egger’s test for 1-month overall survival**

Visual assessment and the Egger’s test does not implicate publication bias.

**Figure S21 Cummulative ranking curves and SUCRA for methodology-based evaluation
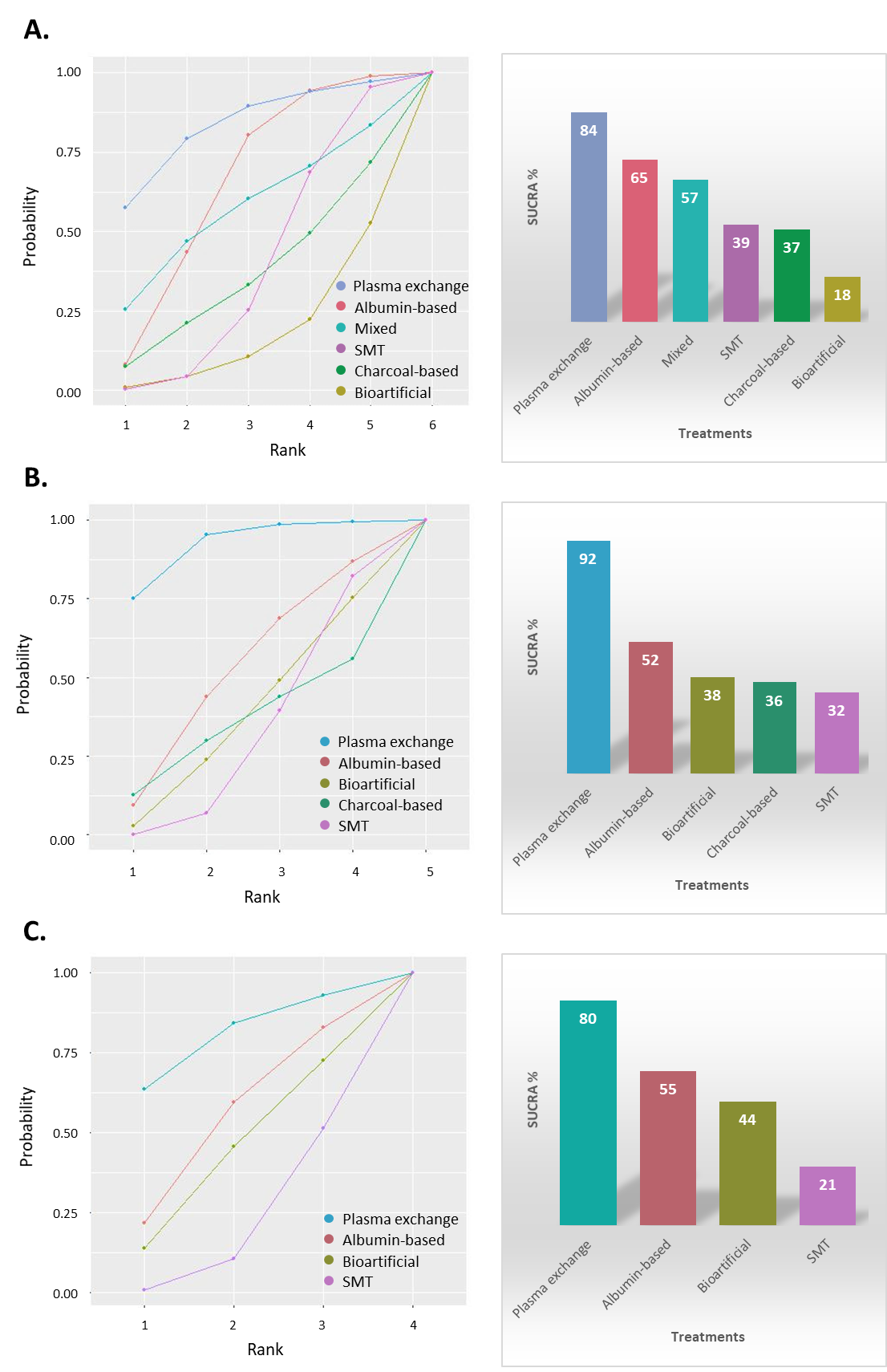
**A. 1-month overall survival B. 3-month overall survival C. 3-month transplant-free survival

Albumin-based: MARS and Prometheus; Mixed: MARS+ plasma exchange; SMT: standard medical therapy; Charcoal-based: BiologicDT; Bioratificial: ELAD

**Figure S22 Methodology-based evaluation league tables**

The league table contains the risk ratios (RR) and credible intervals (CrI) for every possible comparison of the interventions. Events were defined as death (or transplant for TFS) during the follow-up period. Albumin-based: MARS and Prometheus; Mixed: MARS+ plasma exchange; SMT: standard medical therapy; Charcoal-based: BiologicDT; Bioratificial: ELAD
